# Synthetic antimicrobial peptides as enhancers of the bacteriolytic action of staphylococcal phage endolysins

**DOI:** 10.1038/s41598-022-05361-1

**Published:** 2022-01-24

**Authors:** Ana Gouveia, Daniela Pinto, Helena Veiga, Wilson Antunes, Mariana G. Pinho, Carlos São-José

**Affiliations:** 1 Research Institute for Medicines (iMed.ULisboa), Faculdade de Farmácia da Universidade de Lisboa, Av. Prof. Gama Pinto, 1649-003 Lisbon, Portugal; 2 Instituto de Tecnologia Química e Biológica António Xavier, Universidade Nova de Lisboa, Av. da Républica, 2780-157 Oeiras, Portugal; 3 Unidade Militar Laboratorial de Defesa Biológica e Química (UMLDBQ), Instituto Universitário Militar, Centro de Investigação da Academia Militar (CINAMIL), Av. Dr. Alfredo Bensaúde, 1849-012 Lisbon, Portugal

**Keywords:** Antimicrobials, Applied microbiology, Bacteriophages, Cellular microbiology

## Abstract

Bacteriophage endolysins degrade the bacterial cell wall and are therefore considered promising antimicrobial alternatives to fight pathogens resistant to conventional antibiotics. Gram-positive bacteria are usually considered easy targets to exogenously added endolysins, since their cell walls are not shielded by an outer membrane. However, in nutrient rich environments these bacteria can also tolerate endolysin attack if they keep an energized cytoplasmic membrane. Hence, we have hypothesized that the membrane depolarizing action of antimicrobial peptides (AMPs), another attractive class of alternative antibacterials, could be explored to overcome bacterial tolerance to endolysins and consequently improve their antibacterial potential. Accordingly, we show that under conditions supporting bacterial growth, *Staphylococcus aureus* becomes much more susceptible to the bacteriolytic action of endolysins if an AMP is also present. The bactericidal gain resulting from the AMP/endolysin combined action ranged from 1 to 3 logs for different *S. aureus* strains, which included drug-resistant clinical isolates. In presence of an AMP, as with a reduced content of cell wall teichoic acids, higher endolysin binding to cells is observed. However, our results indicate that this higher endolysin binding alone does not fully explain the higher susceptibility of *S. aureus* to lysis in these conditions. Other factors possibly contributing to the increased endolysin susceptibility in presence of an AMP are discussed.

## Introduction

Antibiotic resistance is currently a major threat to global health and economy, with catastrophic scenarios anticipated if efficient control actions are not taken^1–3^. One of the measures to tackle the problem relies on the development of alternative antimicrobials capable of acting on drug-resistant bacteria, preferentially with new modes of action that minimize the emergence of resistance. Among such alternatives in the pipeline are endolysins^4^, which are enzymes (enzybiotics) that destroy the bacterial cell wall (CW)^5^.

Endolysins are produced by bacteriophages (phages), viruses that infect bacteria. An endolysin and a holin define the minimal tool set that double-stranded DNA phages use to lyse host bacteria for virion progeny release at the end of infection^6^. The holin forms “holes” in the cytoplasmic membrane that cause cell death through dissipation of the membrane proton-motive force (PMF)^7^. In addition, for phages employing the so-called canonical lysis model, the holin holes also provide the conduit for passage of the cytoplasm-accumulated endolysin to the CW compartment. Once there, endolysins degrade the peptidoglycan, the major structural component of the CW, and at least for Gram-positive bacteria this is usually sufficient to cause osmotic cell lysis^7,8^.

Application of endolysins as enzybiotics against Gram-positive bacteria is usually considered facilitated, since these lack the outer membrane that in Gram-negative bacteria and mycobacteria hinders enzybiotic access to the CW^8,9^. However, despite lacking this CW protecting barrier, a few studies have shown that Gram-positive bacteria can also restrict or tolerate endolysin attack to certain extent. This was observed for several bacterial species/endolysin pairs, with endolysin tolerance being favored in media that supported bacterial growth, but abolished upon membrane PMF collapse by the holin or by ionophores mimicking its action^10–12^. Hence, the hallmark was that to counteract endolysin lytic action, cells needed to be in a competent energetic state, i.e., with an operational PMF. This phenomenon seems to recapitulate the natural context of phage infection, where endolysins only act after the holin-mediated PMF dissipation (lysis mechanisms reviewed in Ref.^8^).

The mechanisms linking the PMF to the bacterial capacity to restrict endolysins lytic action are still not understood, although the importance of the PMF for control of the activity of some bacterial lytic enzymes (autolysins) is well-documented. One of the bacterial CW components proposed to respond to the ionic changes induced by PMF collapse are wall teichoic acids (WTA), which are abundant anionic polymers bound to the CW of many Gram-positive bacteria. Some studies have presented WTA as key elements restricting autolysin activity in a PMF-dependent way (Refs.^13,14^ and references therein). Other studies have shown that WTA can be responsible for restricting access of autolysins and endolysins to certain regions of the CW, therefore coordinating their spatial distribution^15,16^. More recently, it was proposed that WTA can protect bacteria from the attack of lytic enzymes by hindering their binding to the CW, with this shielding effect being stronger when cells are in rich nutritional media^17–19^. For other enzymes however, binding to WTA appears to be a requirement for lytic activity^20^.

Current knowledge indicates therefore that susceptibility of Gram-positive bacteria to endolysins lytic action can be highly dependent on the cells physiologic/energetic state, something that could impact the efficacy of the lytic enzymes as antibacterials^21^. We have reasoned that the holin key role in sensitizing bacteria to endolysins could be substituted by the action of antimicrobial peptides (AMPs). AMPs are produced by virtually all living organisms as part of the defense mechanisms against bacteria (and other microbes), and frequently their action involves permeabilization of the bacterial cytoplasmic membrane, with consequent PMF collapse^22,23^. AMPs have been also regarded as promising alternatives to conventional antibiotics due to their particular mechanism of action and immunomodulatory features^23^.

By using a PMF-disrupting AMP and a staphylococcal endolysin as models, we have explored the capacity of the AMP to enhance the lytic power of the endolysin against the high priority, Gram-positive pathogen *S. aureus*^24^, including methicillin resistant *S. aureus* (MRSA) clinical strains, under growth supporting conditions. We have also focused on the mechanism(s) by which the AMP facilitated endolysin lytic action, considering its multiple effects at the cellular level. Lastly, we have briefly investigated the role of WTA in endolysin tolerance.

## Results

### Lytic action of endolysin Lys11 is enhanced in the presence of the AMP R8K

**Figure 1:**
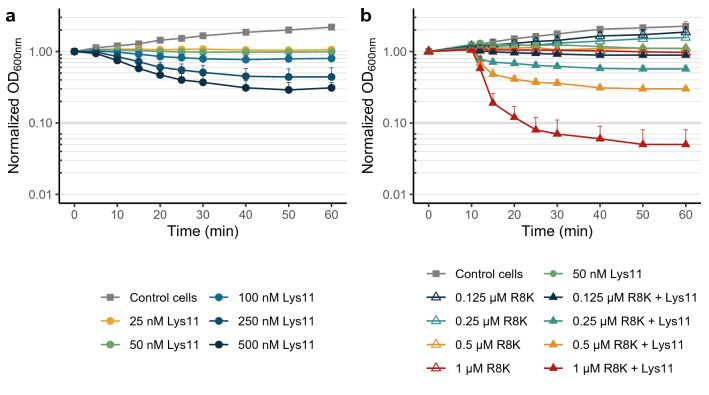
Lytic activity of the endolysin Lys11 in absence or presence of the AMP R8K. (**a**) Log phase cells of strain RN4220 were collected in pre-warmed fresh TSBca, and lysis monitored by taking OD measurements (OD_600nm_) after addition of the indicated Lys11 concentrations or of endolysin buffer (Control cells curve). (**b**) Log phase cells of strain RN4220 collected in TSBca were treated for 10 min with the indicated concentrations of R8K. Then, 50 nM of Lys11 were added and cell lysis evaluated spectrophotometrically. AMP solvent and endolysin buffer were added to the Control cells. Each curve represents means ± standard deviation from at least 3 independent experiments. For clarity, only mean + standard deviation is represented.

We showed previously that the *S. aureus* ability to counteract the attack of the phage ϕ11 endolysin Lys11 was clearly diminished in presence of the PMF-dissipating agent gramicidin^12^. To have a better understanding of *S. aureus* capacity to resist Lys11-mediated lysis, cells in rich culture media (TSB) were treated with different concentrations of Lys11 and cell lysis monitored by taking optical density (OD) measurements over time. These assays were carried out in the presence of 0.5 mM CaCl_2_ (TSBca) as we have recently observed an enhancing effect of calcium ions on the lytic activity of Lys11, as described for related enzymes^25,26^. The two lowest enzyme concentrations tested (25 and 50 nM, about 1.4 and 2.8 μg/ml, respectively) produced no significant cell lysis after 60 min contact, although they could arrest *S. aureus* growth (Fig. 1a). In the same conditions, the other tested enzyme concentrations (100, 250 and 500 nM) reduced culture’s OD roughly by 20, 50 and 70%, respectively.

Next, we tested if *S. aureus* cells treated with an AMP would become more prone to Lys11-mediated lysis. For these assays we selected the peptide SMAP-29 (K^8^), for simplicity hereafter designated R8K, which corresponds to a low toxicity version of the natural cathelicidin SMAP-29 (sheep myeloid antimicrobial peptide of 29 amino acids)^27^, which in turn carries an amphipathic α-helical segment responsible for membrane permeabilization^28^. Exponentially growing *S. aureus* cells recovered in fresh TSBca were pre-treated for 10 min with different concentrations of R8K (0.125 to 1 μM) and then supplemented with 50 nM of Lys11, which we have shown previously to cause no lysis (green curve in Fig. 1a). The results showed that *S. aureus* cells became more susceptible to Lys11-mediated lysis as the R8K concentration increased (Fig. 1b). When cells were pre-treated with 1 μM R8K, which corresponded to its minimal inhibitory concentration (MIC) in our experimental conditions, 50 nM of Lys11 were sufficient to clear the culture after about 10 min of contact (time point 20 min in Fig. 1b, ~ 90% cell lysis according to OD measurements). Although at the MIC the R8K peptide caused no visible lysis in absence of Lys11 (Fig. 1b), determination of colony forming units (CFU) revealed between 1 to 1.5 log reduction of cell viability resulting solely from the AMP action (Supplementary Fig. S1). By using the PMF-sensitive probe DiSC_3_(5), we have confirmed that R8K at its MIC caused abrupt membrane depolarization (Supplementary Fig. S2), which should be responsible for the observed CFU reduction. The determination of CFU counts for the range of R8K concentrations tested suggested a positive correlation between the bactericidal effect of R8K and its capacity to sensitize cells to Lys11 lytic activity (see below).

**Figure 2:**
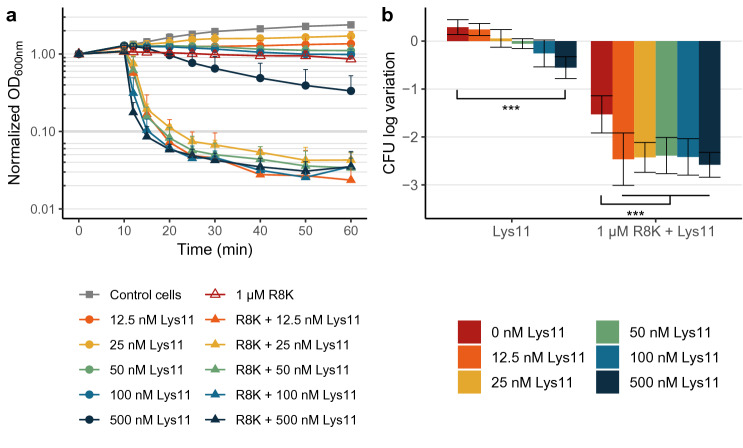
The enhanced Lys11-mediated bacteriolysis in presence of R8K results in a bactericidal gain. (**a**) Log phase cells of strain RN4220 collected in TSBca were treated for 10 min with 1 μM R8K or with the peptide solvent. After this treatment, the indicated concentrations of Lys11 were added to the samples and cell lysis evaluated by OD_600nm_ measurements. AMP solvent and endolysin buffer were added to the Control cells. (**b**) Cell viability at time point 60 min of panel (**a**) was evaluated by CFU counts. For each condition, the results are represented as the log variation of CFU/ml relatively to the cell input. The data of each condition represent means ± standard deviation from at least 3 independent experiments. Asterisks indicate a significant difference of CFU log variations according to one-way ANOVA, followed by Bonferroni post hoc test (***P < 0.001). For clarity, only mean + standard deviation is represented in (**a**).

The peptide R8K clearly enhanced the bacteriolytic effect of Lys11, but we wondered if the combined action of the two agents translated into a bactericidal gain when compared to the AMP alone. Cells pre-treated with 1 μM R8K were challenged with different Lys11 concentrations (from 12.5 to 500 nM) and cell lysis and viability monitored as before. Cultures were rapidly and massively lysed in presence of R8K and Lys11 (Fig. 2a), even at the lowest enzyme concentration (12.5 nM), whereas the endolysin alone essentially reproduced the absent to moderate lysis observed in Fig. 1a. In fact, when the AMP was present, no obvious dose response to Lys11 could be observed unless the endolysin concentration was progressively lowered from 12.5 to 0.78 nM (~ 0.04 μg/ml), which still could lyse at least 90% of the cultures within 60 min (Supplementary Fig. S3). The results indicated that R8K-treated *S. aureus* cells were efficiently lysed with minute amounts of Lys11. Reversing the order of R8K and Lys11 additions or treating cultures with both agents at the same time essentially produced the same results, apart from a slight and expected delay on the onset of lysis (due to the time R8K needed to exert its action) and a minor decrease of the lysis rate, especially for the combinations with the lower Lys11 concentrations (Supplementary Fig. S4).

Cell viability for all conditions at time point 60 min (Fig. 2a) was assessed by CFU counts. As observed before, the peptide reduced cell viability by almost 1.5 log units (Fig. 2b), whereas the endolysin could only produce a discernible impact on cell viability at the highest concentration (500 nM, ~ 0.5 log reduction). In presence of the two agents, CFU counts were reduced by up to 2.5 log units, and basically independently of Lys11 concentration, which agreed with the observed dose independence in lysis at the tested concentrations. Overall, the combination R8K/Lys11 produced a gain in cell death of 1 and 2 log units compared to the isolated action of the AMP and endolysin, respectively, which suggested a synergistic effect resulting from the joint action of the two agents. This AMP effect in sensitizing bacteria to endolysin attack was similarly verified for other endolysin/AMP combinations (Supplementary Fig. S5) using the endolysin LysK^29^ and the AMP vAMP 059, the latter of which was previously shown to kill *S. aureus* due to its membrane-targeting properties^30^.

### AMP R8K sensitizes MRSA strains to endolysin attack

**Figure 3:**
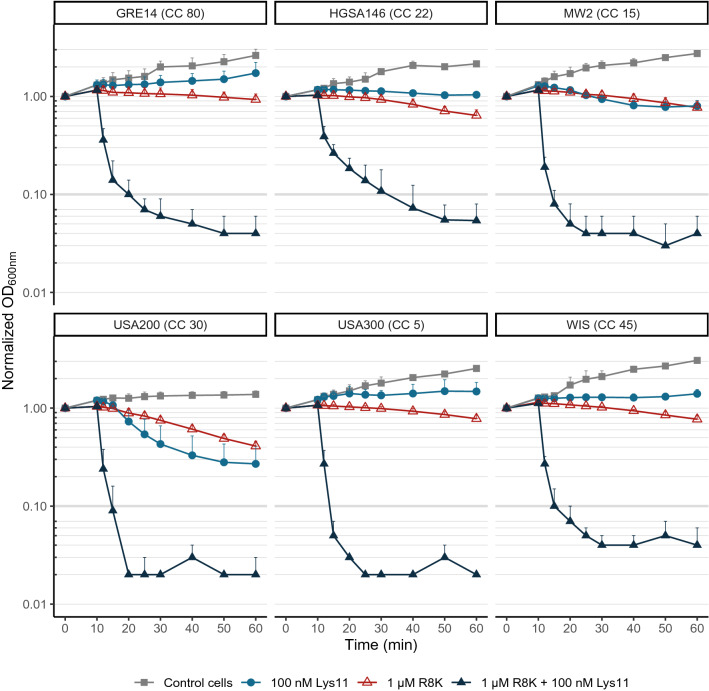
The AMP R8K makes MRSA strains highly susceptible to Lys11-mediated lysis. Log phase cells of the indicated MRSA strains were collected in TSBca and treated for 10 min with 1 μM R8K or with the peptide solvent. After this treatment, 100 nM of Lys11 were added to samples and cell lysis evaluated by OD_600nm_ measurements. AMP solvent and endolysin buffer were added to the Control cells. Each curve represents means ± standard deviation from at least 3 independent experiments. For clarity, only mean + standard deviation is represented. Strain designations are indicated above each graph. *CC* clonal complex.

**Figure 4:**
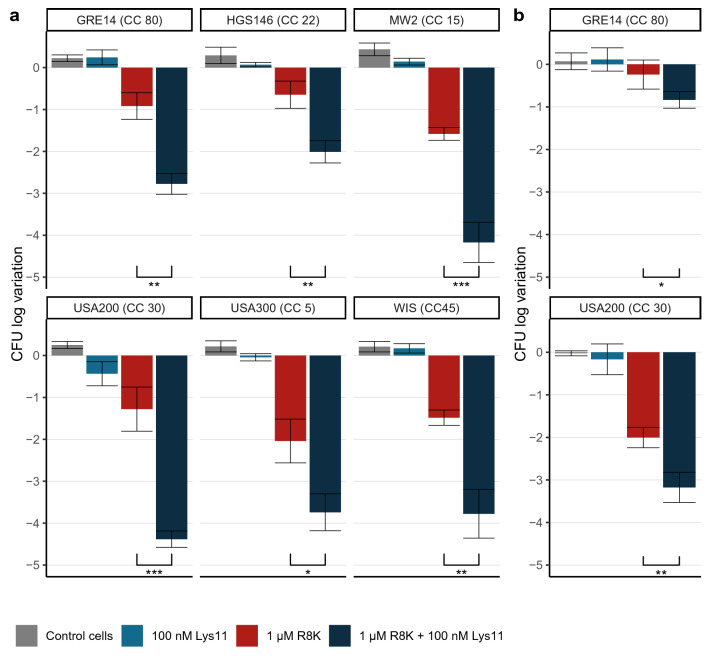
The combined action of R8K and Lys11 results in enhanced bactericidal action against MRSA strains. (**a**) Cell viability of MRSA strains at time point 60 min of Fig. 3 was evaluated for each condition by CFU counts. (**b**) The bactericidal effect of the agents was similarly evaluated against cells of strains GRE14 and USA200 in human blood serum (cell input of ~ 1 × 10^6^ CFU/ml). The results are represented as the log variation of CFU/ml relatively to the cell input. The data represent means ± standard deviation from at least 3 independent experiments. Asterisks indicate a significant difference of the CFU log reduction between the conditions R8K and R8K + Lys11 within each strain, according to independent samples *t*-Tests (*P < 0.05; **P < 0.01; ***P < 0.001).

The experiments from the previous section were carried out with the laboratory *S. aureus* strain RN4220. Therefore, we wanted to check if similar results were obtained with clinically relevant MRSA strains. To that end, the bacteriolytic and bactericidal assays described above were carried out with 6 MRSA stains representative of 6 clonal complexes^31^, using 1 μM R8K and/or 100 nM Lys11. The results confirmed none or very weak lysis as result of the individual action of the two agents, except for strain USA200 that showed lysis with either agent, although higher with the endolysin (Fig. 3). In agreement with the previous results, R8K-treated cultures of these strains were completely cleared in 10 min or less after endolysin addition, apart from strain HGSA146 that took ~ 10 additional minutes to reach an OD reduction of at least 90%. Remarkably, enumeration of cell counts at time point 60 min showed that the bactericidal gain resulting from the combined action of the two agents was substantially higher for some of the clinical strains, with cell death improved by ~ 1.5 log units in strains GRE14 and HGSA146 and up to 3 log units in strains MW2 and USA200, when compared to the R8K induced lethality (Fig. 4a). Overall, the results demonstrated the synergistic bacteriolytic and bactericidal action of the two agents against MRSA strains.

AMPs and endolysins may be rapidly degraded, eliminated or have their activity inhibited in complex biological environments, like those found in the human body. Particularly, several AMPs were shown to be susceptible to the presence of salts and/or components present in the human blood serum^32^. The bactericidal action of R8K and Lys11 was assayed individually and in combination in human blood serum against log phase cells of the clinical MRSA strains GRE14 and USA200. These strains were selected because they showed one of the lowest and highest bactericidal gains as result of the combined R8K/Lys11 action in culture medium (~ 1.5 and ~ 3 log units, respectively, Fig. 4a). The assay conditions were as in the experiments of Figs. 3 and 4a, except that target cells were lowered to a more clinically meaningful concentration (~ 1 × 10^6^ instead of ~ 1 × 10^8^ CFU/ml). In the serum, the endolysin at 100 nM caused none or minor reduction of the CFU counts of GRE14 and USA200, respectively (Fig. 4b), in agreement with its action in culture medium (Fig. 4a). Strain USA200 however showed to be much more susceptible to the AMP in these conditions than GRE14 (2 versus 0.2 log reduction, respectively, Fig. 4b). The joint action of the two agents resulted in a bactericidal gain of ~ 1 and ~ 0.5 log units against USA200 and GRE14, respectively. Thus, the bactericidal gain in the serum for the two strains was about threefold lower than that observed in the culture medium. In conclusion, although the combined action of the agents appeared not as effective as in the TSBca, overall, the results in human blood serum were in line with those observed in the bacterial culture medium for the two strains.

### *Staphylococcus aureus* cells treated with R8K retain their cellular morphology

**Figure 5:**
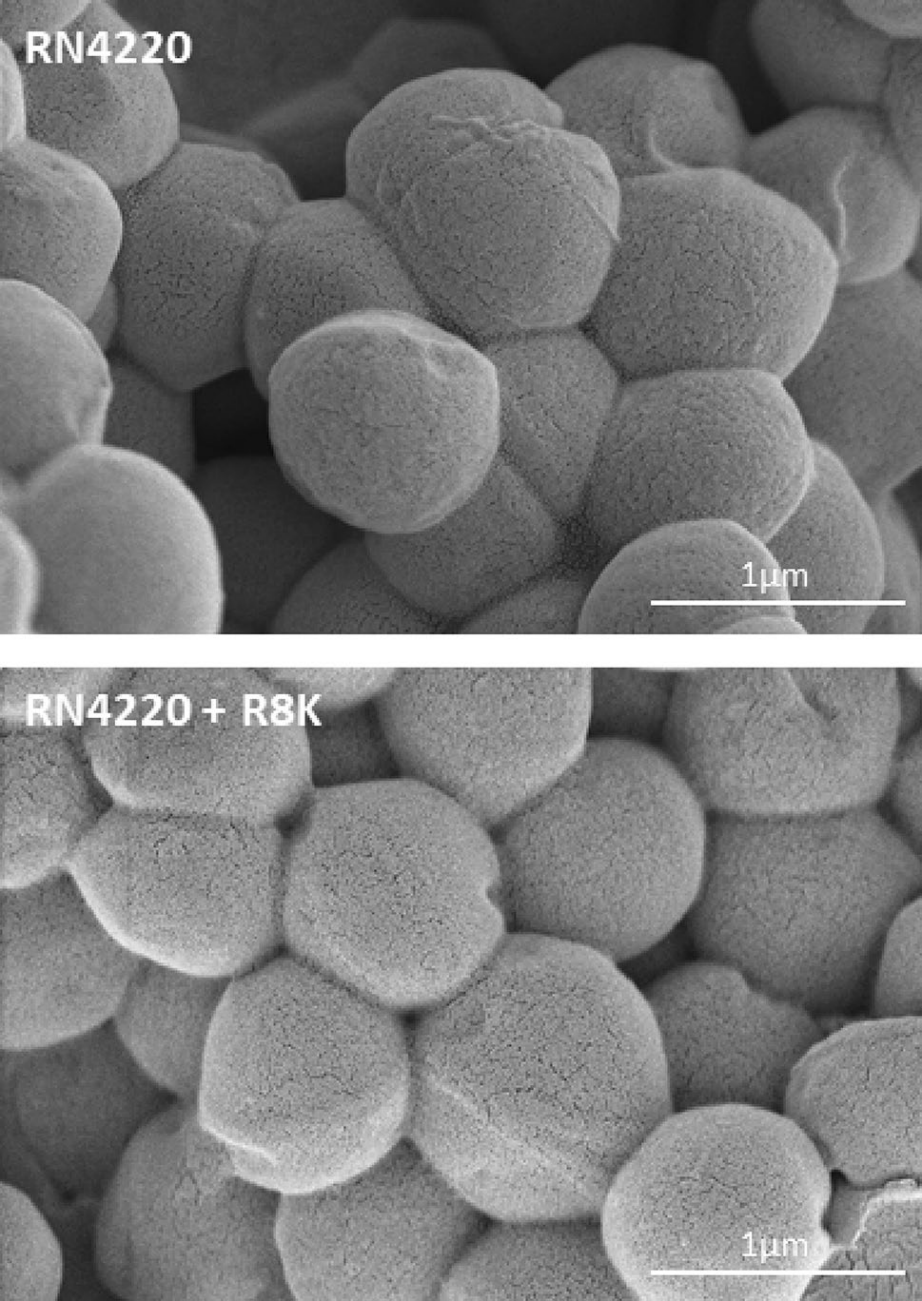
The AMP R8K does not change the overall morphology and cell surface features of *S. aureus*. Log phase cells of strain RN4220 collected in TSBca were treated for 10 min with 1 μM R8K (bottom panel) or with the AMP solvent (top panel) and then visualized by Scanning Electron Microscopy. Scale bar corresponds to 1 μm.

Motivated by the results described above, we set to investigate the mechanisms by which R8K facilitated Lys11 lytic action. Under certain conditions, some AMPs, specially at or above the MIC, can induce major damages to the bacterial cell envelope, as observed for some SMAP-29 congeners (reviewed in Ref.^28^). Such action on the cell envelope could explain the R8K effect in making the *S. aureus* CW much more vulnerable to Lys11 attack. Although our previous assays indicated that no substantial cell lysis resulted from the isolated action of R8K at the MIC (Figs. 1b, 2a), we wanted to check if the AMP was causing major changes in the cell surface and shape of staphylococcal cells. *S. aureus* cells were treated or not with R8K, in the same conditions of the previous experiments, and visualized by ultra-resolution scanning electron microscopy (SEM). We could not identify any obvious changes regarding cell shape and cell surface integrity when comparing untreated with R8K-treated cells (Fig. 5). Hence, there was no indication that the observed R8K/Lys11 synergistic effect could be related with major R8K-induced CW damages.

### Peptide R8K enhances Lys11 binding to *S. aureus* cells without affecting the pattern of endolysin distribution on the cellular surface

Some studies have linked endolysin tolerance to a deficient binding of the lytic enzymes to cells, caused by WTA (see “Introduction”). Therefore, we wondered if the R8K action promoted an increase of Lys11 binding to *S. aureus* cells, which could favor the enzyme’s lytic activity. To test this, we have fused the enhanced green fluorescent protein (eGFP) to a non-lytic form of the endolysin and quantified the amount of fusion protein bound to untreated and R8K-treated cells.

Lys11 carries two catalytic domains, CHAP and Amidase_2, followed by a cell binding domain of the SH3_5 family (Supplementary Fig. S6a). The CHAP peptidase domain was shown to account for almost the entire peptidoglycan-degrading activity of the endolysin in vitro^33^. The Amidase_2 domain of the highly related endolysin LysSA12 was also reported to contribute negligibly to CW lytic activity but to greatly enhance endolysin binding to *S. aureus* cells^34^. We have observed a similar role of the Amidase_2 domain in Lys11 binding to cells (data not shown) and have therefore constructed the fusion eGFP-Amidase_2-SH3_5. We confirmed that the fusion, for simplicity henceforth designated eGFP-Ami11-CBD11, had no lytic activity in our assay conditions, both in absence and presence of the AMP R8K (Supplementary Fig. S6b).

**Figure 6:**
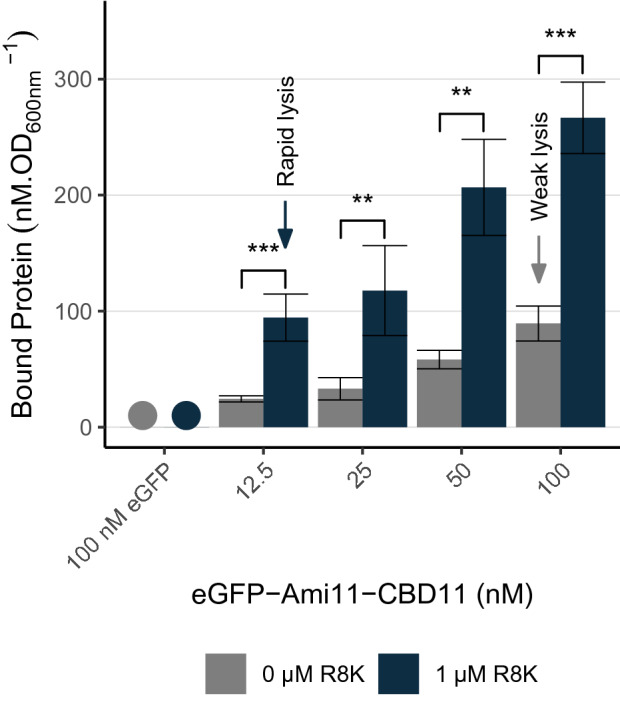
Endolysin binding to *S. aureus* cells is enhanced upon R8K treatment. Log phase cells of strain RN4220 collected in TSBca were treated for 10 min with 1 μM R8K or with the peptide solvent. Following this treatment, the indicated concentrations of eGFP-Ami11-CBD11 were added to the samples and further incubated for 10 min. Cells were washed to discard unbound fluorescent protein and the amount of eGFP-Ami11-CBD11 associated to cells quantified by fluorometry (data of each sample corrected for the final OD_600nm;_ see “Materials and methods” for details). As a control of unspecific binding, a similar assay was run in parallel with 100 nM eGFP, which consistently originated very low values of cell-associated fluorescence that were incompatible with reliable quantification (noted with circles). For each tested eGFP-Ami11-CBD11 concentration, the amount of bound protein to R8K-treated cells was three- to four-fold higher. The arrows highlight two conditions showing similar amounts of eGFP-Ami11-CBD11 bound to cells, which produced very different lysis phenotypes in a Lys11 equivalent context (see Fig. 2a). The data represent means ± standard deviation from at least 3 independent experiments. Asterisks indicate a significant difference between the amount of eGFP-Ami11-CBD11 bound to untreated cells (0 μM R8K) and to R8K-treated cells, for each protein concentration, according to independent samples *t*-Tests (**P < 0.01; ***P < 0.001).

As in the experiments of Fig. 2, *S. aureus* cells were treated or not with 1 μM R8K. Next, eGFP-Ami11-CBD11 was added to cells at different concentrations (12.5 to 100 nM). After 10 min contact, cells were washed to remove free eGFP-Ami11-CBD11 and the amount of protein associated to the cell population estimated from fluorescent measurements (see “Materials and methods” for details). The results showed that eGFP-Ami11-CBD11 binding to R8K-treated cells was favored for all tested concentrations, with the amount of bound protein being three- to four-fold higher when compared to untreated cells (Fig. 6). Cells from the conditions 12.5 nM eGFP-Ami11-CBD11 with and without R8K were also visualized by fluorescence microscopy and the intensity of the fluorescent signal associated to cells quantified. This approach also indicated higher binding of eGFP-Ami11-CBD11 to R8K-treated cells, with the fluorescence signal associated with *S. aureus* cells being 1.6 ± 0.2-fold higher when compared to the condition without R8K.

In these assays we used eGFP-Ami11-CBD11 as a proxy of Lys11 binding to cells. We should note though that besides lacking lytic activity, the mass of the fluorescent fusion was about 10 kDa higher than that of the native endolysin. These differences could affect the capacity of eGFP-Ami11-CBD11 to penetrate through the CW and should be taken into account. Despite this, it is worth noticing that in absence of R8K the amount of bound protein after incubation with 100 nM eGFP-Ami11-CBD11 was not significantly different from that observed after incubation of R8K-treated cells with 12.5 nM eGFP-Ami11-CBD11 (arrows in Fig. 6). Yet, with R8K + 12.5 nM Lys11 we observed rapid cell lysis (orange triangles in Fig. 2a), whereas with 100 nM of the endolysin alone only a very weak lytic effect was produced (light blue circles in Fig. 2a). Thus, it appears that for similar amounts of bound endolysin, very different lysis phenotypes are observed depending on the presence or absence of R8K. Hence, although probably contributing to improve lysis, the increased affinity of the endolysin to R8K-treated cells does not seem sufficient to explain the highly enhanced Lys11 lytic activity in presence of the AMP, suggesting that the AMP stimulates endolysin lytic action by additional means. Therefore, we questioned if R8K could be changing the subcellular localization of Lys11, by allowing for example access to CW sites otherwise inaccessible. To answer this question, we have analyzed, by super-resolution structured illumination fluorescence microscopy, the pattern of eGFP-Ami11-CBD11 localization on the CW of untreated and R8K-treated cells. The analysis revealed that eGFP-Ami11-CBD11 distributed around the CW surface, with preferential accumulation at cell junctions, irrespective of the presence or absence of the AMP (Supplementary Fig. S7). Therefore, the much higher lytic action of Lys11 against R8K-treated cells does not seem to result from an alteration of the endolysin binding pattern to S*. aureus* cells.

### *Staphylococcus aureus* treatment with antibiotics and susceptibility to Lys11

**Figure 7:**
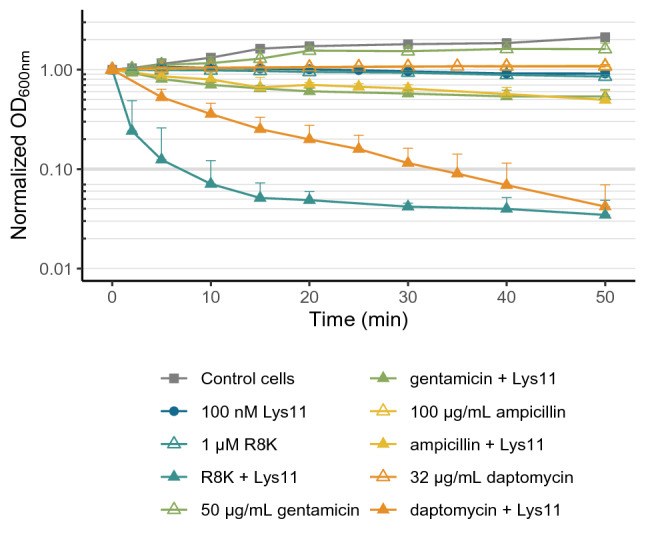
Endolysin activity against cells treated with antimicrobials with different cellular targets. Log phase cells of strain RN4220 collected in TSBca were treated with 1 μM R8K, 50 μg/ml gentamicin, 100 μg/ml ampicillin or 32 μg/ml daptomycin (10-min treatment for R8K and 30-min for the antibiotics). Then, 100 nM of Lys11 were added and cell lysis evaluated spectrophotometrically. Antimicrobial solvents and endolysin buffer were added to the Control cells. Each curve represent means ± standard deviation from at least 3 independent experiments. For clarity, only mean + standard deviation is represented.

We showed that in addition to favoring Lys11 binding to the cells, the AMP R8K also caused cell death, most probably due to its membrane disruption action. In fact, the results suggested that the R8K capacity to sensitize *S. aureus* cells to Lys11 lysis correlated with its bactericidal action (Fig. 1b and Supplementary Fig. S1). Therefore, we questioned if other agents capable of killing or inhibiting *S. aureus* growth to the same extent as R8K, but exhibiting a different mode of action, would similarly improve Lys11-mediated bacteriolysis. In our assay conditions, treating cultures for 30 min with 50 μg/ml of gentamicin, a bactericidal antibiotic inhibiting protein synthesis, here used at 100 times the reported MIC for strain RN4220^35^, resulted in a CFU reduction of 1.23 ± 0.38 log units, which was close to the killing effect of the AMP (~ 1.5 log). Further 50 min incubation reduced cell counts by 3.76 ± 0.09 log units. When *S. aureus* cultures pre-treated 30 min with gentamicin were subjected to the action of 100 nM Lys11 during 50 min, only partial (~ 50% OD reduction) and rather slow lysis was observed when compared to the abrupt and extensive lysis obtained in presence of the AMP (Fig. 7). Therefore, simple cell death cannot reproduce the strong effect of R8K as enhancer of endolysin lytic action.

Some drugs interfering with the cytoplasmic membrane function and its capacity to generate the PMF can also result in CW synthesis impairment^36,37^. To evaluate if a potential inhibitory action of R8K on peptidoglycan synthesis contributed to lysis susceptibility, we have tested the effect of the antibiotic ampicillin, which inhibits CW peptidoglycan synthesis and cell division, on Lys11-meadiated lysis. Under our experimental setting, cell growth halting occurred in less than 30 min with the addition of 100 μg/ml ampicillin (data not shown), which corresponded to about 128 times the reported ampicillin MIC for strain RN4220^38^. Thirty minutes incubation with ampicillin produced only a slight reduction of cell viability (0.23 ± 0.17 log units). Additional incubation until timepoint 80 min (mimicking the 30 min antibiotic incubation plus the 50 min of incubation with the lysin) caused a 0.78 ± 0.18 log reduction of CFU counts. As observed for gentamicin, the addition of Lys11 30 min after ampicillin treatment produced only modest and delayed lysis (Fig. 7), indicating that specific inhibition of peptidoglycan synthesis cannot make cells vulnerable to Lys11 lysis to the extent that R8K does.

The previous results supported that PMF-dissipation promoted by R8K was important to *S. aureus* sensitization to Lys11. Thus, we wondered if an antibiotic sharing this mode of action would be able to enhance the endolysin lytic effect. One such antibiotic is daptomycin^39^, which at 32 times its MIC (32 μg/ml) reduced RN4220 CFU counts by 1.43 ± 0.72 and 3.09 ± 0.63 log units, after 30- and 80-min contact, respectively. In contrast to what we observed with the previous antibiotics, the 30 min pre-treatment with daptomycin clearly made strain RN4220 much more vulnerable to Lys11 lytic action, although not as efficiently as with R8K (Fig. 7). Interestingly, the lower capacity of daptomycin in sensitizing cells to Lys11-mediated lysis seemed to correlate with a much slower and less extensive membrane depolarization produced by the antibiotic (Supplementary Fig. S2). Overall, the results supported the notion that PMF-dissipation is a key event to increase *S. aureus* susceptibility to Lys11, and that agents provoking fast and strong membrane depolarization are the best sensitizers.

### WTA are involved in *S. aureus* tolerance to Lys11

One aspect we started to explore in this work was the identification of cellular determinants of tolerance to endolysins. WTA were previously implicated in tolerance based on their capacity to hamper the binding of some lytic enzymes to target bacteria^17–19^, but for some endolysins they were also shown to work as the ligands required for specific recognition and binding to the CW^20^. We have therefore studied the impact of WTA on *S. aureus* susceptibility to Lys11 lytic action and on endolysin binding to cells. WTA synthesis can be severely inhibited in *S. aureus* with low concentrations of tunicamycin, without significantly affecting peptidoglycan synthesis and cell growth. For several *S. aureus* strains, including the laboratory strain RN4220 used here, this specific effect can be obtained with as low as 50 ng/ml of tunicamycin^40,41^.

**Figure 8:**
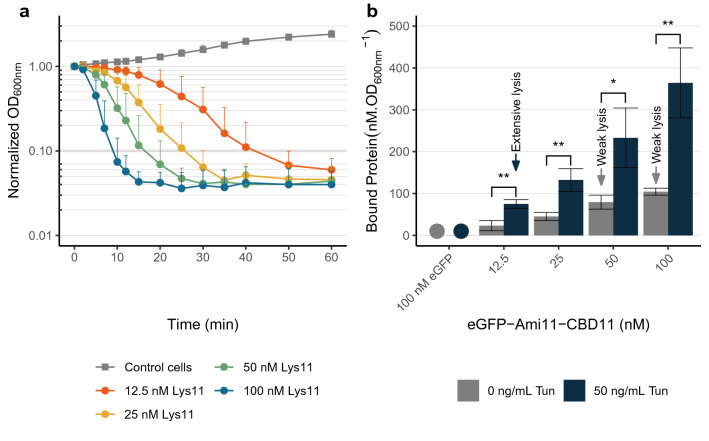
Endolysin binding and lytic activity are enhanced against *S. aureus* cells grown in presence of tunicamycin. (**a**) Log phase cells of strain RN4220 grown in presence of 50 ng/ml tunicamycin were collected in TSBca and then incubated with the indicated concentrations of Lys11 or endolysin buffer (Control cells curve), and lysis monitored by OD_600nm_ measurements. (**b**) Log phase cells of strain RN4220 grown in absence or presence of tunicamycin (Tun) were collected in TSBca, incubated for 10 min with the indicated concentrations of eGFP-Ami11-CBD11, and the amount of fluorescent protein bound to cells quantified as in Fig. 6. As for the assays of Fig. 6, we could not measure any significant binding of eGFP to cells (noted with circles). For each tested eGFP-Ami11-CBD11 concentration, the amount of protein bound to tunicamycin-treated cells was 3 to 3.5-fold higher. The arrows highlight conditions showing similar amounts eGFP-Ami11-CBD11 bound to cells, but which produced very different lysis phenotypes in a Lys11 equivalent context (see panel a and Fig. 1a). The data represent means ± standard deviation from at least 3 independent experiments. Asterisks indicate a significant difference between the amount of eGFP-Ami11-CBD11 bound to cells grown in absence (0 ng/ml) or presence of tunicamycin (Tun), for each protein concentration, according to independent samples *t*-Tests (*P < 0.05; **P < 0.01). For clarity, only mean + standard deviation is represented in (**a**).

Cells grown in presence of tunicamycin for three generations were challenged with different concentrations of Lys11 (12.5 to 100 nM) to evaluate their susceptibility to lysis. Tunicamycin-treated cells revealed to be significantly more susceptible to Lys11 bacteriolysis, with the highest endolysin concentration (100 nM) decreasing 90% of the culture OD in less than 10 min, and the lowest (12.5 nM) in about 40 min (time points 10 and 40 min in Fig. 8a).

To assess the effect on Lys11 binding, cells grown in the presence of tunicamycin were brought into contact with the same concentration range of eGFP-Ami11-CBD11 as in Fig. 6. The fluorescent protein bound 3 to 3.5-fold more effectively to cells grown in presence of tunicamycin (Fig. 8b). Yet, for the same reason explained above, this increased binding seems insufficient to explain the higher bacteriolytic activity of Lys11 towards cells lacking WTA, since for similar amounts of bound protein (arrows in Fig. 8b), obvious lysis is only observed with tunicamycin-treated cells (compare orange curve in Fig. 8a with green and light blue curves in Fig. 1a). Hence, WTA role in endolysin tolerance should go beyond the simple shielding effect in binding. Also, for reasons that are still unknown, we conclude that WTA cannot fulfill their inhibitory role when cells are treated with R8K.

## Discussion

The use of endolysins as antibacterials is based on the idea that the lytic enzymes can efficiently lyse and kill bacteria when added from outside if access to the CW is granted. However, recent studies have shown that this “lysis from without”, nicely revealed for a Gram-positive pathogen about 20 years ago^42^, may be hampered under certain physiological and growth conditions of target bacteria. The general observation is that energized bacteria actively growing in rich media may tolerate endolysins attacking from without, with the extent of tolerance depending on the endolysin/bacterium pair. Considering the possibility that this capacity of bacteria to fightback the attack of lytic enzymes may negatively impact their performance in a therapeutic context^21^, it is crucial to understand the tolerance mechanisms, and how to overcome them, to maximize endolysins enzybiotic potential.

The first studies describing endolysin tolerance recognized a relationship between the PMF and bacterial susceptibility to the lytic enzymes^10–12^. Such direct or indirect role of the PMF in controlling endolysin tolerance is not surprising, if we consider that in the phage lytic cycle endolysins only act after the abrupt, holin-mediated collapse of the PMF^8^. In addition, it has long been established the role of the PMF in the regulation of autolysins^13,14^, which are peptidoglycan-cleaving enzymes structurally and functionally related to endolysins. Therefore, endolysins might also be subjected to the same PMF-dependent mechanisms that control autolysins activity in the bacterial CW. All these observations prompted us to search for fast acting PMF dissipaters with the potential to be used as enhancers of endolysin lytic action in clinical contexts. One obvious possibility were AMPs.

In this work we show that when cultures of *S. aureus* suffer the action of AMPs, endolysins can produce much faster and/or extensive cell lysis (Fig. 2a and Supplementary Fig. S5). In the assay conditions, Lys11 at the maximum concentration (500 nM) had only a modest impact on cell viability (~ 0.5 log reduction), while the MIC of R8K killed between 1 to 1.5 log units. When acting together, the two agents produced a lethality gain of 1 to 3 log units relatively to the AMP alone, depending on the tested *S. aureus* strain (including MRSA). Therefore, although the drastic effect of the AMP in stimulating endolysin-mediated bacteriolysis was essentially transversal to all tested stains, the bactericidal gain resulting from the combined action of the two agents was more variable. Such variation may reflect differences between strains regarding the composition, structure and/or modifications of their cell envelope, which potentially could impact AMP and endolysin activities. The R8K MIC showed minor variation among the strains used in this work, ranging between 1 μM (strains RN4220, HGS146, MW2, USA200 and USA300) and 2 μM (GRE14 and WIS). Thus, the relative susceptibility to R8K does not seem to explain the distinct susceptibility of the strains to the R8K/Lys11 combined action (Fig. 4). It will be interesting to study in in vivo models of infection if the AMP/endolysin co-treatment results in therapeutic advantage when compared to the isolated action of the two antibacterials.

Different peptides, including AMPs like SMAP-29, have been fused to endolysins targeting Gram-negative bacteria. With this modification, the endolysins (called Artilysins) gained the capacity to cross the outer membrane of Gram-negative bacteria (reviewed in Ref.^43^). Inspired by this work, Rodríguez-Rubio et al. showed that fusing a polycationic peptide to the endolysin λSa2lys increased its anti-streptococcal activity, probably by improving the enzyme’s affinity to the cell surface^44^. Other studies have explored synergisms resulting from the simultaneous action of CW lytic enzymes and agents damaging the cytoplasmic membrane. Desbois and Coote^45^ showed that lysostaphin was synergistically bactericidal in combination with the lantibiotic nisin and lipopeptide antibiotics (colistin, daptomycin and polymyxin B). It was also found that the combination of endolysins with daptomycin, a PMF dissipator antibiotic^39^, resulted in better antibacterial activity against *S. aureus* and *Streptococcus pneumonia*, in vitro and in vivo, when compared to the agents isolated action^46–48^. The authors proposed that the peptidoglycan-degrading activity of the endolysins could be promoting fastest and more efficient membrane insertion of daptomycin. Considering the results presented here, it is also possible that the PMF-depolarizing action of the antibiotic has contributed to endolysins lytic activity. Interestingly, endolysins were also shown to synergize with antibiotics having distinct modes of action, like those inhibiting the CW and protein synthesis^49,50^. Such synergy though was not obvious for the tested antibiotics in our assay conditions, considering their poor stimulatory effect on Lys11 lytic activity (Fig. 7). In any case, the described examples support that synergism between endolysins and other agents deserve further exploration as a source of new antibacterial strategies.

Two key questions that still require further elucidation are: (i) what are the determinants and mechanisms responsible for tolerance, and (ii) what are the exact mechanisms by which AMPs abolish tolerance and strongly stimulate endolysin lytic activity. In agreement with previous reports involving endolysins and autolysins^17–19,29^, we verified that WTA were important for *S. aureus* tolerance to Lys11. Based on the observation that in rich media WTA can hinder the binding of peptidoglycan-degrading enzymes to bacterial cells, namely to *S. aureus*, it was proposed that tolerance relied on a WTA-dependent shielding of the CW^17–19^. We have also observed such inhibitory role of WTA on Lys11 binding. However, our data suggest that this shielding effect explains only partially the function of this CW polymer in the control of lytic enzymes (Fig. 8b). It is possible that in absence of WTA endolysins can not only bind more, but also get deeper into the CW matrix. WTA have been also implicated in the localization and regulation of the peptidoglycan synthetic machinery, influencing for example the level of peptidoglycan cross-linking^51^. Perturbation of these processes due to the lack of WTA could also turn cells more susceptible to endolysins attack.

As stated above, PMF collapse in many Gram-positive bacteria, including *S. aureus*, activates autolysins^13^. A link has been proposed between the ΔpH component of the PMF and WTA in the control of autolytic activity, in which the WTA capacity to retain protons would create an acidic environment in the CW that inhibited autolysins^14^. In this scenario, the PMF-dissipating action of R8K would also cause “deprotonation” of WTA, eventually creating a less acidic and more favourable milieux for Lys11 lytic activity. We can also raise the hypothesis of a destabilization of WTA resulting from the interaction between R8K and WTA. This interaction is likely to occur if we consider the AMP positive charge (between + 8 and + 9 at culture medium pH) and the anionic nature of WTA^52^. In fact, interaction of cationic AMPs with anionic polymers of the cell envelope, like WTA, is frequently a key step for the subsequent AMP insertion in the cytoplasmic membrane^53^. In any event, it was interesting to note that R8K retained the capacity to stimulate Lys11-mediated lysis of *S. aureus* lacking WTA (Supplementary Fig. S8), suggesting that the AMP action does not depend on the presence of WTA in the CW.

Interfering with WTA and/or peptidoglycan synthesis with peptides or conventional antibiotics can promote susceptibility to lysis, namely by deregulation of the endogenous autolytic enzymes^54,55^. However, such possible effect resulting from R8K action does not seem to be contributing significantly to lysis in our assay conditions, given the modest effect of ampicillin in sensitizing cells to Lys11 lytic activity (Fig. 7). In any case, our results indicate that all the discussed potential pathways by which the PMF and WTA could be contributing to endolysin tolerance, somehow become non-functional or ineffective upon *S. aureus* treatment with the AMP R8K.

Based on the exposed above and in our results, we tend to believe that the AMP role in abolishment of endolysin tolerance is a multifactorial process, which has as key event the PMF dissipation. In addition to promoting endolysin binding to cells, R8K-mediated collapse of the PMF will certainly change the CW ionic environment, the level of protonation of WTA and their function, and eventually cause deregulation of peptidoglycan synthesis. All these events could collectively compromise the cell capacity to cope with lytic enzymes. Although our SEM analysis did not reveal any major damage in the *S. aureus* cell surface, one cannot exclude the possibility that the AMP also inflicts undetected vulnerabilities that facilitate endolysin access deeper in the CW peptidoglycan mesh. Further studies will be important to elucidate the endolysin tolerance mechanisms and why they suddenly collapse upon AMP action.

## Materials and methods

### Bacterial strains and growth conditions

Unless stated otherwise, *Escherichia coli* strains were routinely grown at 37 °C in LB medium, under aerated conditions. The plasmid expressing eGFP-Ami11-CBD11 was recovered in *E. coli* strain XL1-Blue MRF’ (Stratagene). *E. coli* strain CG61, a BL21 derivative that produces phage T7 RNA polymerase upon thermal induction^56^, was used to overexpress Lys11, eGFP and eGFP-Ami11-CBD11, whereas strain BL21-Gold(DE3) was used to overproduce LysK^29^. For selection of plasmid-bearing cells, LB medium was supplemented with 100 µg/ml ampicillin and/or 40 µg/ml kanamycin. Specific culture conditions for protein expression are explained below. The *S. aureus* lab strain RN4220^57^ and the MRSA clinical strains^31^ were grown in tryptic soy broth (TSB) medium at 37 °C under aerated conditions. For impairment of WTA synthesis, TSB was supplemented with 50 ng/ml tunicamycin. To minimize variations on RN4220 growth rates, TSB cultures were initiated by diluting 100-fold standardized frozen inocula, which were prepared as follows. Cells were grown until an optical density at 600 nm (OD_600nm_) of 0.8, collected by centrifugation, and resuspended in half-volume of fresh TSB supplemented with 16% glycerol. Cell suspensions were stored at − 80 °C as 200 µl aliquots.

### Expression and purification of endolysins

The overexpression and purification of Lys11 was carried out as described before^12^, with minor modifications. After overnight growth at 28 °C, *E. coli* CG61 expressing Lys11 was 100-fold diluted in LB medium buffered with 0.1 M phosphate buffer pH 7.2 and supplemented with 0.5 M d-sorbitol. For preparation of this medium, twofold concentrated LB with 1 M d-sorbitol and a 0.2 M sodium phosphate buffer solution were prepared and sterilized separately, and then mixed in a 1:1 ratio to reconstitute the medium. Cultures were grown at 28 °C until OD_600nm_ of about 0.5, after which protein synthesis was induced by incubating cultures for 30 min at 42 °C in a shaking water bath. The water bath temperature was rapidly decreased to 16 °C with ice and cultures further incubated for 14–16 h. Cells were recovered by centrifugation (8000*g*, 15 min, 4 °C) and resuspended in 1/50 volume of lysis buffer (50 mM Hepes, 500 mM NaCl, 0.1% triton, 10% glycerol, 1 mM DTT, 50 mM Imidazole, pH 7.0) supplemented with 1 × Complete Mini EDTA-free Protease Inhibitor Cocktail (Roche Applied Science), 10 mM MgCl_2_ and 10 µg/ml DNase I. Cells were disrupted by sonication (Vibra Cell, Sonic Materials) with 7 to 10 bursts of 15 s (amplitude 50%, pulse 5, 20–30 W), with 45 s pauses between bursts. Extracts were maintained on ice during sonication. Crude protein extracts were cleared by centrifugation (30,000*g*, 30 min, 4 °C) and purification was performed by metal chelate affinity chromatography as described previously^12^. Lys11 pure fractions were exchanged to an imidazole-free buffer (same composition of lysis buffer but without imidazole) using HiTrap™ or HiPrep 26/10 desalting columns (GE Healthcare). Production and purification of LysK was carried out as for Lys11 except for the following two changes: (i) an *E. coli* BL21-Gold(DE3) derivative harboring pET21a::*lysK*^29^ was grown until late exponential phase (OD_600nm_ ~ 1). At this stage, protein production was induced with 0.5 mM IPTG and cells incubated in a shaking water bath set to 16 °C for 14–16 h; (ii) lysis buffer composition was 50 mM Hepes, 300 mM NaCl, 30% glycerol, 50 mM imidazole, pH 8.0) with the same supplements. Protein fractions were quantified by the Bradford method (Bio-Rad Laboratories), using bovine serum albumin (BSA) as standard. The enzymes were divided in small aliquots and kept at − 80 °C until use.

### Antimicrobial peptides

The peptide SMAP-29 (K^8^), here designated R8K, with the amino acid sequence RGLRRLGKKIAHGVKKYGPTVLRIIRIAG, corresponds to a derivative of SMAP-29 carrying the single R8K substitution^27^. R8K was supplied by the UPF Peptide Synthesis Facility, Universitat Pompeu Frabra, Barcelona, Spain, with acetylated N-terminus and amidated C-terminus (95% HPLC purity). Peptide vAMP 059, with the sequence NWKKWWQVFYTVV^30^, was provided by Miguel Castanho Lab (IMM, Universidade de Lisboa, Lisboa, Portugal) with an amidated C-terminus and a free amine N-terminus (> 95% HPLC purity). Stock solutions of 2 mM were prepared from lyophilized peptides in sterile ultra-pure water and stored at − 20 °C as 20-μl aliquots.

### AMPs minimal inhibitory concentration (MIC)

*Staphylococcus aureus* strain RN4220 was grown until an OD_600nm_ of 0.8. Cultures were then diluted in fresh TSB to a cell density of ~ 1 × 10^6^ colony forming units per milliliter (CFU/ml) and distributed into the wells of a 96-well microtiter plate (50 μl per well). Serial twofold dilutions of the AMPs were prepared in TSB and 50 μl added to cells (100 μl final volume). The plates were incubated at 37 °C for 24 h after which they were observed. The recorded MIC corresponded to the lowest AMP concentration inhibiting *S. aureus* growth. MICs were determined in triplicate for each AMP.

### Bacteriolytic, bactericidal and membrane depolarization assays

Unless stated otherwise, *S. aureus* cells were grown until mid-exponential phase (OD_600nm_ ~ 0.4), collected by centrifugation (6000*g*, 7 min), and resuspended in half volume of fresh TSB supplemented with 0.5 mM CaCl_2_ (TSBca) prewarmed at 37 °C. The final OD_600nm_ of cell suspensions was ~ 0.8, which corresponded to a cell density of ~ 1 × 10^8^ CFU/ml. The ability of endolysins to cause cell lysis, either when acting alone or after the indicated treatments with different agents (AMPs, gentamicin, ampicillin or daptomycin), was evaluated under static conditions at 37 °C. Cells impaired in WTA synthesis were similarly prepared, except that TSB/TSBca was supplemented with 50 ng/ml tunicamycin. Lysis was monitored by taking OD_600nm_ measurements at defined time points after endolysin addition, over a period of up to 60 min. Growth controls were similarly prepared and received the equivalent volumes of the agents solvents.

The bactericidal activity of the endolysin, alone or in combination with other agents, was assessed in terms of the impact on cell viability, expressed as CFU/ml. CFU counts were determined simultaneously with the bacteriolytic assays, for the indicated time points and conditions, by diluting samples in phosphate-buffered saline (PBS) and plating on tryptic soy agar (TSA) plates. Changes in cell viability were expressed as the log variation of CFU/ml relatively to the initial cell input. The bactericidal activity of the endolysin Lys11, isolated or in combination with the AMP R8K, was also assessed in human blood serum (from human male AB plasma, Sigma-Aldrich, Cat. No. H4522). Target cells at OD_600nm_ ~ 0.8 were prepared as described above and diluted 100-fold in the blood serum (~ 1 × 10^6^ CFU/ml) before challenge with the agents. After 50- or 60-min incubation at 37 °C, CFU counts were determined as described above.

The PMF-dissipation action of R8K and daptomycin was confirmed using the membrane potential-sensitive dye DiSC_3_(5) (Sigma-Aldrich, Cat. No. 43608) as described elsewhere^58^. Briefly, cells from mid-exponential phase cultures of *S. aureus* strain RN4220 were collected by centrifugation, washed with assay buffer (5 mM HEPES, 20 mM glucose, pH 7.2), and resuspended in the same buffer supplemented with 100 mM KCl and 0.5 mM CaCl_2_ to a final OD_600nm_ of 0.05. Cells were incubated for 15 min at 37 °C and then distributed through wells of a black microtiter plate (Greiner Bio-One, Cat. N. 655076). DiSC_3_(5) was added to a final concentration of 0.5 µM and the plates incubated in the dark at 37 °C for 30 min to enable dye uptake by the cells and fluorescence quenching. Fluorescence measurements were made using excitation and emission wavelengths of 622 and 672 nm, respectively, to confirm stable fluorescence readings (Varioskan LUX Multimode, ThermoFisher Scientific). The test agents were then added at the indicated concentrations and fluorescence variation measured for additional 50 min. Controls with free DiSC_3_(5) dissolved in supplemented assay buffer were similarly analyzed to monitor interferences of the agents with the dye.

### Scanning electron microscopy

Five milliliter sample cultures of *S. aureus* RN4220 at OD_600nm_ ~ 0.8 in TSBca were obtained as described above and incubated with the peptide R8K at its MIC (1 μM) or with its solvent for 10 min at 37 °C. Cells were pelleted (3000*g*, 15 min) and resuspended in 1 ml of 0.1 M sodium cacodylate buffer, pH 7.2. Suspensions were again centrifuged (10,000*g*, 7 min) and cells fixed by resuspension in fixation solution (0.4% glutaraldehyde, 4% paraformaldehyde, 0.1 M sodium cacodylate buffer) and incubation for 60 min at room temperature. Cells were washed three times with 0.1 M sodium cacodylate buffer and dehydrated by covering the cellular pellets with a graded ethanol series (50, 70, 90 and 100%, 10 min each). Cells were overlaid with tert-butyl alcohol prewarmed at 30 °C, incubated for 60 min at room temperature, and kept overnight at − 20 °C for complete solidification of the tert-butyl alcohol. Samples were dried in a centrifugal vacuum concentrator for 10 min at 37 °C (Genevac™ miVac Centrifugal Concentrator). The dried samples were then mounted in golden sputtered lamellae and sputtered with gold to achieve a final golden layer of ~ 3 nm thickness (Cressington 108 golden sputter). The lamellae were attached to an aluminum stub using a double face copper tape and observed in a Hitachi SU8010 scanning electron microscope, with a beam acceleration of 1 kV, for a working distance of 1.6 mm in beam deceleration mode, to achieve a final resolution of 1.3 nm.

### Construction of the eGFP-endolysin fusion and cell binding assays

eGFP-Ami11-CBD11 gene fusion was assembled by overlap-extension polymerase chain reaction (OE-PCR) using the high-fidelity NZYProof DNA polymerase (NZYTech—Genes & Enzymes) and previously described plasmids for *lys11* and *eGFP* templates^12,59^. The Ami11-CBD11 moiety corresponded to a C-terminal fragment of Lys11 starting at residue P_149_ (GenBank AAL82281.2) and included the putative linker region connecting the CHAP and Amidase_2 domains. The amplified eGFP-Ami11-CBD11 coding sequence carried 5′ and 3′ N*coI* and X*maI* restriction sites, respectively, which allowed its cloning in the expression vector pIVEX2.3d that produced the fusion with an hexahistidine C-terminal tag (Roche Applied Science). A construct expressing the hexahistidine-tagged eGFP was similarly generated. The recombinant plasmids were confirmed by sequencing and transformed into *E. coli* strain CG61 (see above).

For production of eGFP and eGFP-Ami11-CBD11, the CG61 derivatives were grown in LB medium at 28 °C until an OD_600nm_ of 0.8, after which protein production was induced by temperature up-shift as described above for Lys11. After induction, cultures were transferred to an incubator at 37 °C and grown for additional 180 min. Cells were recovered by centrifugation and resuspended in 1/50 volume of lysis buffer (50 mM Hepes, 300 mM NaCl, 30% glycerol, 50 mM imidazole, 1 mM DTT, pH 8.0) supplemented as above. Cell disruption, protein purification, quantification and storage were as described for the endolysins.

For cell binding assays, *S. aureus* RN4220 at OD_600nm_ ~ 0.8 in TSBca was prepared as described for the bacteriolytic and bactericidal experiments (see above) and treated or not for 10 min with 1 × MIC R8K (1 μM) at 37 °C. Cell samples of 200 μl were then put into contact with the indicated concentrations of eGFP or eGFP-Ami11-CBD11 and further incubated for 10 min at 37 °C. To remove unbound proteins, samples were centrifuged (6100*g*, 7 min, room temperature), the supernatant carefully discarded, and cells washed with 1 ml PBS. Cells were pelleted again (8500*g*, 5 min, room temperature) and resuspended in 200 μl fresh PBS. The suspensions were transferred to black microtiter plates (Greiner Bio-One, Cat. No. 655076) and fluorescence measured using an excitation and emission wavelengths of 488 and 507 nm, respectively (Varioskan LUX Multimode, ThermoFisher Scientific). OD_600nm_ was also registered. For quantification of bound protein (nM.OD_600nm_^−1^), standard calibration curves were performed for each fluorescent protein. For that, TSB-grown cells at OD_600nm_ ~ 0.4 were washed with PBS and recovered in half volume of the same buffer (OD_600nm_ ~ 0.8). Known concentrations of the fluorescent proteins were serially diluted in this cell suspension and fluorescence measured as above. This procedure accounted for a possible interference of cells in fluorescence measurements. The same protocol was followed to measure the binding to *S. aureus* RN4220 cells grown in the presence of 50 ng/ml tunicamycin (twofold concentrated in fresh TSBca + 50 ng/ml tunicamycin).

### Fluorescence microscopy

For the Super-resolution Structured Illumination Microscopy (SIM) analysis, a *S*. *aureus* RN4220 culture at OD_600nm_ ~ 0.8 in TSBca was prepared as described above and then divided in two samples. Each culture was incubated at 37 °C for 10 min with R8K MIC (1 μM) or with the peptide solvent, and then for 10 min with 12.5 nM eGFP-Ami11-CBD11 at 37 °C. Unbound protein was removed by centrifugation (6100*g*, 7 min, room temperature) and the pellets were carefully washed with 1 ml PBS and resuspended in 20 μl fresh PBS. Cells were placed on a thin layer of 1.2% agarose in PBS mounted on a gene frame and imaged by SIM using an Elyra PS.1 microscope (Zeiss) with a Plan-Apochromat 63 × /1.4 oil DIC M27 objective and a 488 nm laser. Images were captured using a Pco.edge 5.5 camera and reconstructed using ZEN software (black edition, 2012, version 8.1.0.484) based on a structured illumination algorithm.

To quantify the intensity of eGFP-Ami11-CBD11 signal bound to RN4220 cells treated or not with the AMP R8K, a *S*. *aureus* RN4220 culture at OD_600nm_ ~ 0.8 in TSBca was prepared as above and divided in two samples. Each culture was incubated at 37 °C for 10 min with R8K MIC (1 μM) or with the peptide solvent, and then for 10 min with 12.5 nM eGFP-Ami11-CBD11 at 37 °C. One of the cultures was also incubated for 5 min with the DNA dye Hoechst (1 µg/ml) during the eGFP-Ami11-CBD11 incubation period. The samples were then centrifugated (6100*g*, 7 min, room temperature) and the pellets were carefully washed with 1 ml PBS and resuspended in 20 μl fresh PBS. The two samples were then mixed and rapidly placed on a microscope slide covered with a thin layer of 1.2% agarose in PBS and imaged by wide-field fluorescence microscopy using a Zeiss Axio Observer microscope with a Plan-Apochromat 100×/1.4 oil Ph3 objective. Images were acquired with a Retiga R1 CCD camera (QImaging) using Metamorph 7.5 software (Molecular Devices). Four independent experiments were performed, two with R8K-treated cells labeled with Hoechst and the other two with the non-treated culture labeled with Hoechst. The intensity of eGFP-Ami11-CBD11 fluorescence signal bound to the *S. aureus* cells was automatically determined using eHooke cell imaging analysis software^60^. For each experiment, between 1100 to 1500 cells were analyzed and the result is presented as the mean ± standard deviation of the 4 independent experiments.

### Bioinformatics analysis

Protein similarity searches and identification of conserved domains were carried out with BLAST and CDD tools, which are resources of the National Center for Biotechnology Information (https://blast.ncbi.nlm.nih.gov/Blast.cgi), and with Pfam 34.0 (http://pfam.xfam.org/). Protein secondary structures, disordered regions and domain boundaries were analyzed with PSIPRED 4.0, DISOPRED3 and DomPRED (http://bioinf.cs.ucl.ac.uk/psipred/) for prediction of interdomain linker regions.

### Statistical analysis

All data was obtained from repeated assays, with values representing the mean ± standard deviation from 3 to 5 independent experiments. When indicated, statistical significance was evaluated with One-Way ANOVA, followed by Bonferroni post hoc test, or with independent sample *t*-Test.

## Supplementary Information

Supplementary Figures.

## Data Availability

All data are available from the corresponding authors upon reasonable request.

## References

[1] WHO—World Health Organization. Antibiotic Resistance. (2020). https://www.who.int/news-room/fact-sheets/detail/antibiotic-resistance. Accessed 22 Dec 2021.

[2] World Bank. “Drug-Resistant Infections: A Threat to Our Economic Future.” Washington, DC: World Bank. License: Creative Commons Attribution CC BY 3.0 IGO. (2017).http://documents.worldbank.org/curated/en/323311493396993758/pdf/114679-REVISED-v2-Drug-Resistant-Infections-Final-Report.pdf. Accessed 22 Dec 2021.

[3] IACG—Interagency Coordination Group on Antimicrobial Research. No time to wait: securing the future from drug-resistant infections. Report to the secretary-general of the UN. (2019). https://www.who.int/antimicrobial-resistance/interagency-coordination-group/IACG_final_report_EN.pdf?ua=1. Accessed 22 Dec 2021.

[4] Theuretzbacher U, Outterson K, Engel A, Karlén A (2020). The global preclinical antibacterial pipeline. Nat. Rev. Microbiol..

[5] Dams D, Briers Y (2019). Enzybiotics: Enzyme-based antibacterials as therapeutics. Adv. Exp. Med. Biol..

[6] Catalão MJ, Gil F, Moniz-Pereira J, São-José C, Pimentel M (2013). Diversity in bacterial lysis systems: Bacteriophages show the way. FEMS Microbiol. Rev..

[7] Young R (2013). Phage lysis: Do we have the hole story yet?. Curr. Opin. Microbiol..

[8] Fernandes S, São-José C (2018). Enzymes and mechanisms employed by tailed bacteriophages to breach the bacterial cell barriers. Viruses.

[9] Catalão MJ, Pimentel M (2018). Mycobacteriophage lysis enzymes: Targeting the mycobacterial cell envelope. Viruses.

[10] Nascimento JG, Guerreiro-Pereira MC, Costa SF, São-José C, Santos MA (2008). Nisin-triggered activity of Lys44, the secreted endolysin from *Oenococcus oeni* phage fOg44. J. Bacteriol..

[11] Proença D, Leandro C, Garcia M, Pimentel M, São-José C (2015). EC300: A phage-based, bacteriolysin-like protein with enhanced antibacterial activity against *Enterococcus faecalis*. Appl. Microbiol. Biotechnol..

[12] Fernandes S, São-José C (2016). More than a hole: The holin lethal function may be required to fully sensitize bacteria to the lytic action of canonical endolysins. Mol. Microbiol..

[13] Rice KC, Bayles KW (2008). Molecular control of bacterial death and lysis. Microbiol. Mol. Biol. Rev..

[14] Biswas R (2012). Proton-binding capacity of *Staphylococcus aureus* wall teichoic acid and its role in controlling autolysin activity. PLoS One.

[15] Frankel MB, Schneewind O (2012). Determinants of murein hydrolase targeting to cross-wall of *Staphylococcus aureus* peptidoglycan. J. Biol. Chem..

[16] Eugster MR, Loessner MJ (2012). Wall teichoic acids restrict access of bacteriophage endolysin Ply118, Ply511, and PlyP40 cell wall binding domains to the *Listeria monocytogenes* peptidoglycan. J. Bacteriol..

[17] Wu X, Paskaleva EE, Mehta KK, Dordick JS, Kane RS (2016). Wall teichoic acids are involved in the medium-induced loss of function of the autolysin CD11 against *Clostridium difficile*. Sci. Rep..

[18] Wu X, Zha J, Koffas MAG, Dordick JS (2019). Reducing *Staphylococcus aureus* resistance to lysostaphin using CRISPR-dCas9. Biotechnol. Bioeng..

[19] Bhagwat A, Zhang F, Collins CH, Dordick JS (2021). Influence of bacterial culture medium on peptidoglycan binding of cell wall lytic enzymes. J. Biotechnol..

[20] Eugster MR, Haug MC, Huwiler SG, Loessner MJ (2011). The cell wall binding domain of *Listeria *bacteriophage endolysin PlyP35 recognizes terminal GlcNAc residues in cell wall teichoic acid. Mol. Microbiol..

[21] Oliveira H, São-José C, Azeredo J (2018). Phage-derived peptidoglycan degrading enzymes: Challenges and future prospects for in vivo therapy. Viruses.

[22] Haney EF, Mansour SC, Hancock RE (2017). Antimicrobial peptides: An introduction. Methods Mol. Biol..

[23] Mookherjee N, Anderson MA, Haagsman HP, Davidson DJ (2020). Antimicrobial host defence peptides: Functions and clinical potential. Nat. Rev. Drug Discov..

[24] WHO—World Health Organization. Global priority list of antibiotic-resistant bacteria to guide research, discovery, and development of new antibiotics. (2017).https://www.who.int/medicines/publications/WHO-PPL-Short_Summary_25Feb-ET_NM_WHO.pdf. Accessed 22 Dec 2021.

[25] Schmelcher M (2012). *Staphylococcus haemolyticus* prophage ΦSH2 endolysin relies on cysteine, histidine-dependent amidohydrolases/peptidases activity for lysis 'from without'. J. Biotechnol..

[26] Gu J (2014). Structural and biochemical characterization reveals LysGH15 as an unprecedented "EF-hand-like" calcium-binding phage lysin. PLoS Pathog..

[27] Dawson RM, Liu CQ (2011). Analogues of peptide SMAP-29 with comparable antimicrobial potency and reduced cytotoxicity. Int. J. Antimicrob. Agents.

[28] Dawson RM, Liu CQ (2009). Cathelicidin peptide SMAP-29: Comprehensive review of its properties and potential as a novel class of antibiotics. Drug Dev. Res..

[29] Schmelcher M (2015). Evolutionarily distinct bacteriophage endolysins featuring conserved peptidoglycan cleavage sites protect mice from MRSA infection. J. Antimicrob. Chemother..

[30] Dias SA (2017). New potent membrane-targeting antibacterial peptides from viral capsid proteins. Front. Microbiol..

[31] Fernandes S (2012). Novel chimerical endolysins with broad antimicrobial activity against methicillin-resistant *Staphylococcus aureus*. Microb. Drug Resist..

[32] Ageitos JM, Sánchez-Pérez A, Calo-Mata P, Villa TG (2017). Antimicrobial peptides (AMPs): Ancient compounds that represent novel weapons in the fight against bacteria. Biochem. Pharmacol..

[33] Navarre WW, Ton-That H, Faull KF, Schneewind O (1999). Multiple enzymatic activities of the murein hydrolase from staphylococcal phage phi11. Identification of a d-alanyl-glycine endopeptidase activity. J. Biol. Chem..

[34] Son B, Kong M, Ryu S (2018). The auxiliary role of the amidase domain in cell wall binding and exolytic activity of staphylococcal phage endolysins. Viruses.

[35] Hauschild T, Lüthje P, Schwarz S (2005). Staphylococcal tetracycline-MLSB resistance plasmid pSTE2 is the product of an RSA-mediated in vivo recombination. J. Antimicrob. Chemother..

[36] Lunde CS (2009). Telavancin disrupts the functional integrity of the bacterial membrane through targeted interaction with the cell wall precursor lipid II. Antimicrob. Agents Chemother..

[37] Bernal P (2010). Insertion of epicatechin gallate into the cytoplasmic membrane of methicillin-resistant *Staphylococcus aureus* disrupts penicillin-binding protein (PBP) 2a-mediated β-lactam resistance by delocalizing PBP2. J. Biol. Chem..

[38] Fergestad ME (2020). Penicillin-binding protein PBP2a provides variable levels of protection toward different β-lactams in *Staphylococcus aureus* RN4220. Microbiologyopen.

[39] Taylor SD, Palmer M (2016). The action mechanism of daptomycin. Bioorg. Med. Chem..

[40] Swoboda JG (2009). Discovery of a small molecule that blocks wall teichoic acid biosynthesis in *Staphylococcus aureus*. ACS Chem. Biol..

[41] Campbell J (2011). Synthetic lethal compound combinations reveal a fundamental connection between wall teichoic acid and peptidoglycan biosynthesis in *Staphylococcus aureus*. ACS Chem. Biol..

[42] Nelson D, Loomis L, Fischetti VA (2001). Prevention and elimination of upper respiratory colonization of mice by group A streptococci by using a bacteriophage lytic enzyme. Proc. Natl. Acad. Sci. U. S. A..

[43] Briers Y, Lavigne R (2015). Breaking barriers: Expansion of the use of endolysins as novel antibacterials against Gram-negative bacteria. Future Microbiol..

[44] Rodríguez-Rubio (2016). 'Artilysation' of endolysin λSa2lys strongly improves its enzymatic and antibacterial activity against streptococci. Sci. Rep..

[45] Desbois AP, Coote PJ (2011). Bactericidal synergy of lysostaphin in combination with antimicrobial peptides. Eur. J. Clin. Microbiol. Infect. Dis..

[46] Schuch R (2014). Combination therapy with lysin CF-301 and antibiotic is superior to antibiotic alone for treating methicillin-resistant *Staphylococcus aureus*-induced murine bacteremia. J. Infect. Dis..

[47] Watson A, Sauve K, Cassino C, Schuch R (2020). Exebacase demonstrates in vitro synergy with a broad range of antibiotics against both methicillin-resistant and methicillin-susceptible *Staphylococcus aureus*. Antimicrob. Agents Chemother..

[48] Vouillamoz J (2013). Bactericidal synergism between daptomycin and the phage lysin Cpl-1 in a mouse model of pneumococcal bacteraemia. Int. J. Antimicrob. Agents.

[49] Wittekind M, Schuch R (2016). Cell wall hydrolases and antibiotics: Exploiting synergy to create efficacious new antimicrobial treatments. Curr. Opin. Microbiol..

[50] Fang Y (2021). Deimmunized lysostaphin synergizes with small-molecule chemotherapies and resensitizes methicillin-resistant. Antimicrob. Agents Chemother..

[51] Atilano ML (2010). Teichoic acids are temporal and spatial regulators of peptidoglycan cross-linking in *Staphylococcus aureus*. Proc. Natl. Acad. Sci. U. S. A..

[52] Neuhaus FC, Baddiley J (2003). A continuum of anionic charge: Structures and functions of d-alanyl-teichoic acids in gram-positive bacteria. Microbiol. Mol. Biol. Rev..

[53] Anaya-López JL, López-Meza JE, Ochoa-Zarzosa A (2013). Bacterial resistance to cationic antimicrobial peptides. Crit. Rev. Microbiol..

[54] Mellroth P (2012). LytA, major autolysin of *Streptococcus pneumoniae*, requires access to nascent peptidoglycan. J. Biol. Chem..

[55] Homma T (2016). Dual targeting of cell wall precursors by teixobactin leads to cell lysis. Antimicrob. Agents Chemother..

[56] São-José C, Parreira R, Vieira G, Santos MA (2000). The N-terminal region of the *Oenococcus oeni* bacteriophage fOg44 lysin behaves as a bona fide signal peptide in *Escherichia coli* and as a cis-inhibitory element, preventing lytic activity on oenococcal cells. J. Bacteriol..

[57] Kreiswirth BN (1983). The toxic shock syndrome exotoxin structural gene is not detectably transmitted by a prophage. Nature.

[58] Cheng JTJ, Hale JD, Elliot M, Hancock REW, Straus SK (2009). Effect of membrane composition on antimicrobial peptides aurein 2.2 and 2.3 from Australian southern bell frogs. Biophys. J..

[59] Lois C, Hong EJ, Pease S, Brown EJ, Baltimore D (2002). Germline transmission and tissue-specific expression of transgenes delivered by lentiviral vectors. Science.

[60] Saraiva B, Krippahl L, Filipe S, Henriques R, Pinho M (2021). EHooke: A tool for automated image analysis of spherical bacteria based on cell cycle progression. Biol. Imaging..

