# Supplementary material for: Synthetic antimicrobial peptides as enhancers of the bacteriolytic action of staphylococcal phage endolysins
Source: Sci Rep. 2022 Jan 24;12:1245. doi: 10.1038/s41598-022-05361-1 (PMC8786859; doi:10.1038/s41598-022-05361-1)
Supplement: Supplementary file 1 — Supplementary Figures. [file 41598_2022_5361_MOESM1_ESM.pdf]

Supplementary material

Synthetic antimicrobial peptides as enhancers of the bacteriolytic action of staphylococcal phage endolysins

Ana Gouveia[1], Daniela Pinto[1], Helena Veiga[2], Wilson Antunes[3], Mariana G. Pinho[2], Carlos São-José[1]

[1]Research Institute for Medicines (iMed.ULisboa), Faculdade de Farmácia da Universidade de Lisboa, Av. Prof. Gama Pinto, 1649-003 Lisboa, Portugal.

[2]Instituto de Tecnologia Química e Biológica António Xavier, Universidade Nova de Lisboa, Av. da Républica, 2780-157 Oeiras, Portugal

[3]Unidade Militar Laboratorial de Defesa Biológica e Química (UMLDBQ), Instituto Universitário Militar, Centro de Investigação da Academia Militar (CINAMIL), Av. Dr. Alfredo Bensaúde, 1849-012 Lisboa, Portugal

Figure S1. Bactericidal activity of the peptide R8K

Figure S2. R8K causes fast and extensive depolarization of the cytoplasmic membrane

Figure S3. Lysis of R8K-treated *S. aureus* cells in response to decreasing concentrations of Lys11 endolysin

Figure S4. Inverse order or simultaneous addition of R8K and Lys11 also results in enhanced bacteriolysis

Figure S5. Increased endolysin lytic activity in presence of an AMP is observed for other AMP/endolysin combinations

Figure S6. Lys11 and the derived fusion eGFP-Ami11-CBD11

Figure S7. Pre-treatment with the AMP R8K does not alter eGFP-Ami11-CBD11 distribution pattern on *S. aureus* cell surface

Figure S8. R8K stimulates Lys11-mediated lysis of tunicamycin-treated *S. aureus* cells

*For correspondence. E-mail: csaojose@ff.ulisboa.pt; Tel: (+351) 217946420

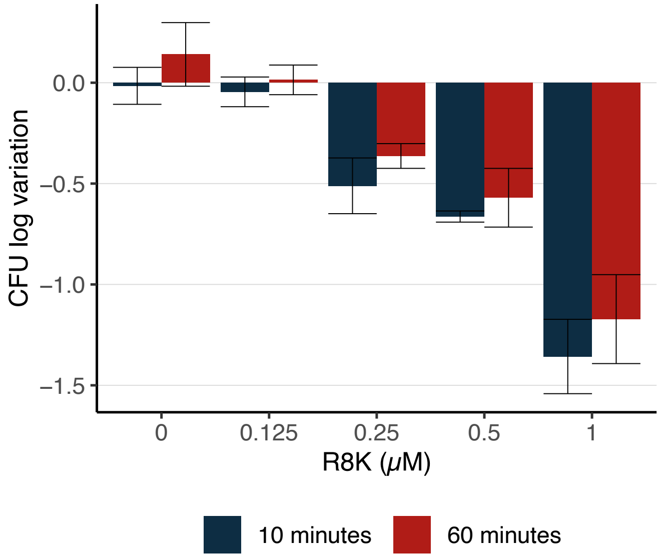

Figure S1. Bactericidal activity of the peptide R8K. Log phase cells of *S. aureus* strain RN4220 were collected in fresh TSBca (~1 x 10^8 CFU/ml) and incubated at 37 ºC for 10 or 60 min with the indicated concentrations of R8K. After the incubation, cell viability was assessed by CFU counts. For each condition, the results are represented as the log variation of CFU/ml relatively to the cell input. The data represent means ± standard deviation from at least 3 independent experiments.

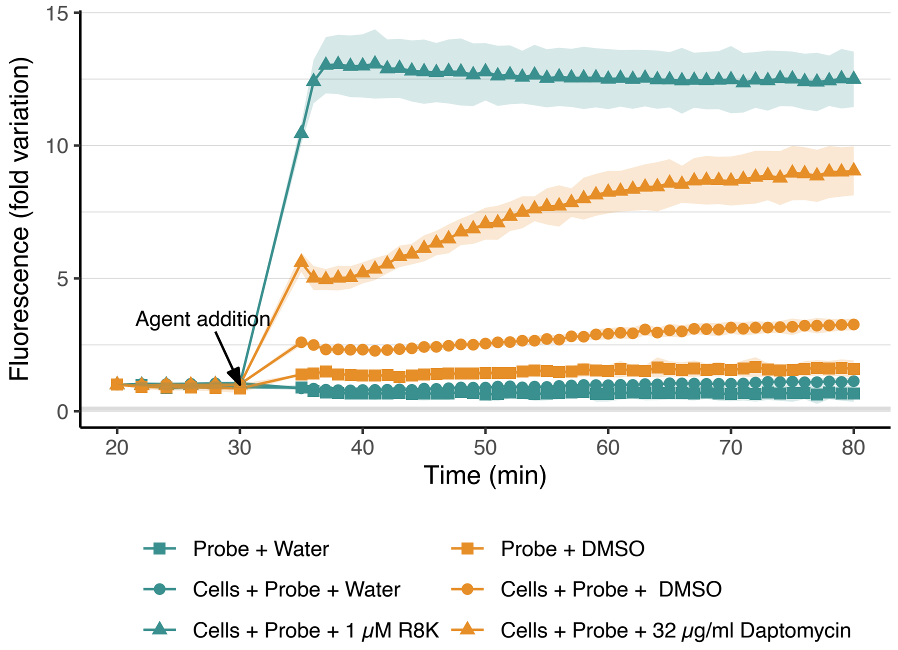

Figure S2. R8K causes fast and extensive depolarization of the cytoplasmic membrane. Cells of *S. aureus* strain RN4220 loaded with the PMF-sensitive probe $DiSC_3(5)$ (see article Materials and Methods) were treated with 1x the MIC of R8K (1 μM) or with 32x the MIC of the membrane-depolarizing antibiotic daptomycin (32 μg/ml). Changes in fluorescence were monitored following the addition of these agents or their solvents (arrow). The effect of the solvents (Water and DMSO) on the fluorescence of free $DiSC_3(5)$ was also controlled. The data of each curve represent means ± standard deviation from at least 3 independent experiments.

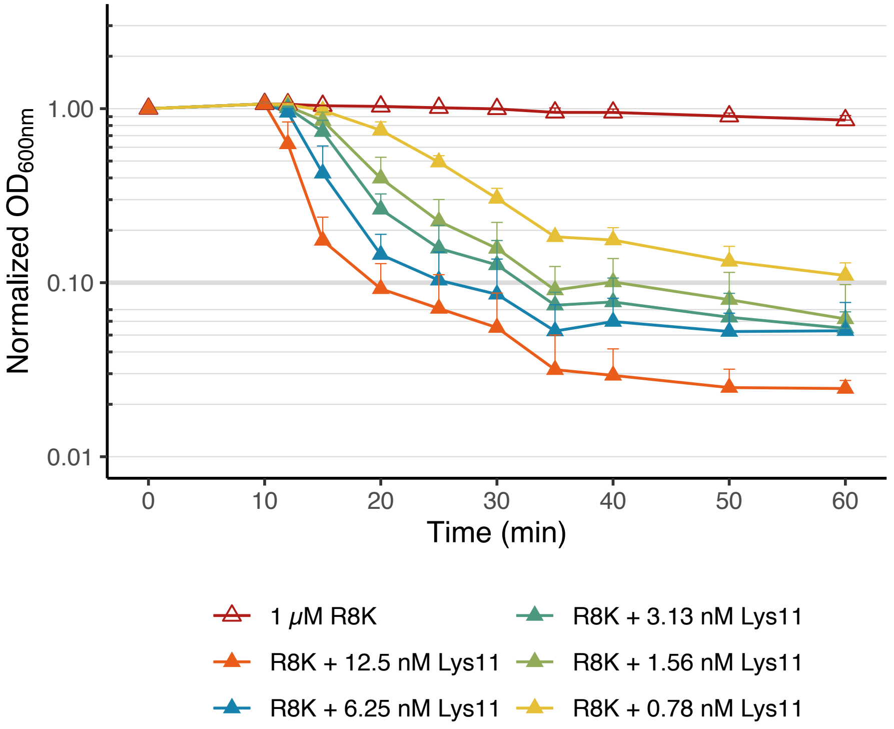

Figure S3. Lysis of R8K-treated *S. aureus* cells in response to decreasing concentrations of Lys11 endolysin. Log phase cells of strain RN4220 collected in TSBca were treated for 10 min with 1 µM R8K, after which the indicated concentrations of Lys11 were added to samples and cell lysis monitored by OD_{600nm} measurements. The data of each curve represent means ± standard deviation from at least 3 independent experiments. For clarity, only mean + standard deviation is represented.

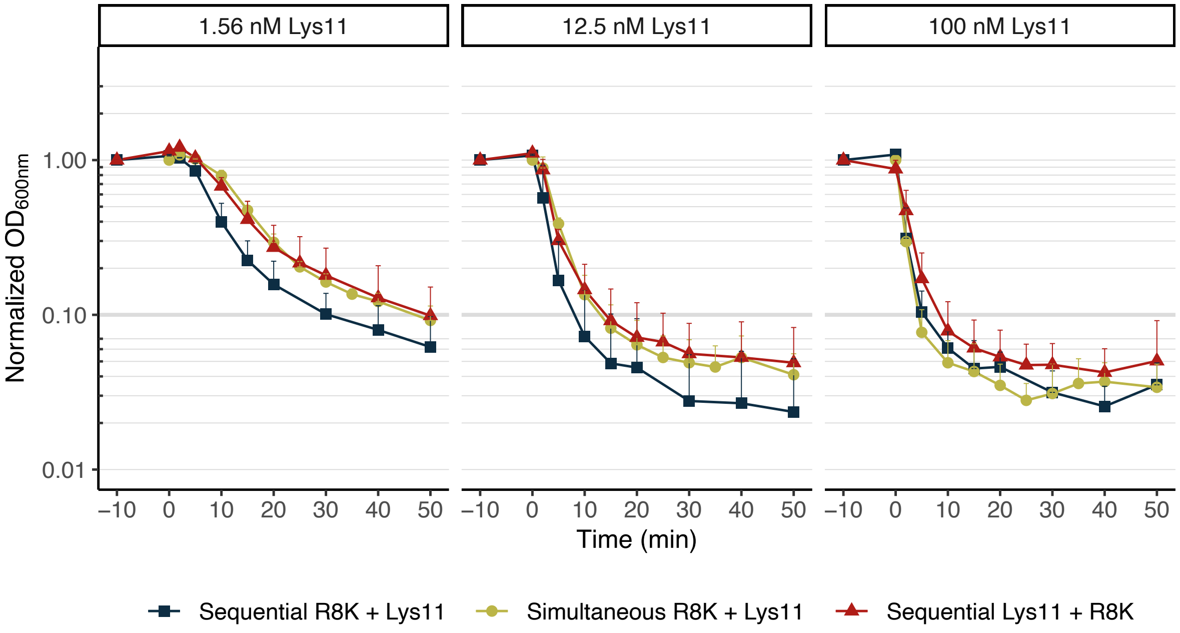

Figure S4. Inverse order or simultaneous addition of R8K and Lys11 also results in enhanced bacteriolysis. Log phase cells of strain RN4220 collected in TSBca were treated sequentially either with 1 µM R8K (10 min) followed by Lys11 (50 min with the indicated concentrations) or with the reverse order (10 min with Lys11 followed by 50 min with 1 µM R8K). In a third condition cells were treated simultaneously with R8K and Lys11 (both added at time 0 min). Cell lysis was monitored by OD_{600nm} measurements in all conditions. The data of each curve represent means ± standard deviation from at least 3 independent experiments. For clarity, only mean + standard deviation is represented.

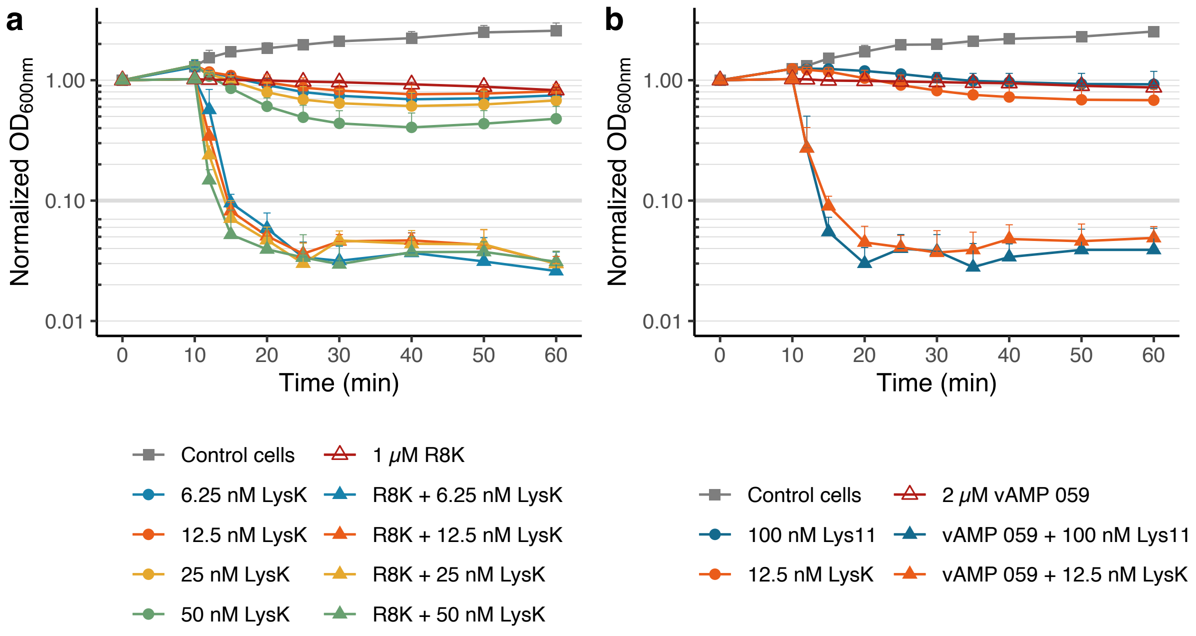

Figure S5. Increased endolysin lytic activity in presence of an AMP is observed for different AMP/endolysin combinations. **(a)** Log phase cells of strain RN4220 collected in TSBca were treated for 10 min with 1 µM R8K or with the AMP solvent. After this treatment, the indicated concentrations of the endolysin LysK were added to samples and cell lysis evaluated spectrophotometrically. AMP solvent and endolysin buffer were added to the Control cells. **(b)** Cells as in (a) were treated for 10 min with 2 µM of the peptide vAMP 059 (MIC) or its solvent. After this period, the indicated concentrations of Lys11 or LysK were added to samples. Cell lysis monitoring and control as in (a). The data of each curve represent means ± standard deviation from at least 3 independent experiments. For clarity, only mean + standard deviation is represented.

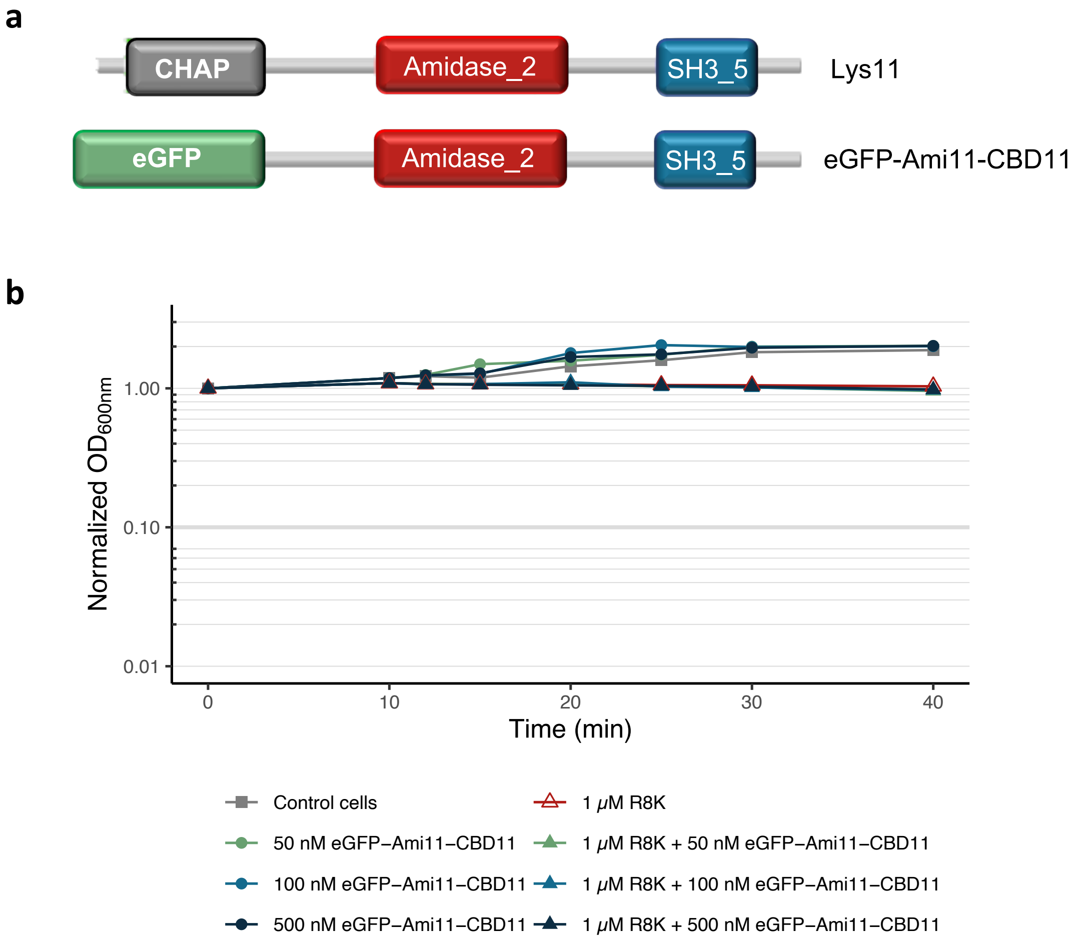

Figure S6. Lys11 and the derived fusion eGFP-Ami11-CBD11. **(a)** Lys11 displays the following domain architecture from N- to C-terminus ([33] and Pfam analysis): a CHAP domain (pfam05257) with peptidase activity, an Amidase_2 domain (pfam01510), and a cell binding domain (CBD) of the SH3_5 family (pfam08460). For construction of the fusion eGFP-Ami11-CBD11, the CHAP domain was substituted by eGFP. **(b)** The fusion eGFP-Ami11-CBD11 does not show lytic activity. Log phase cells of strain RN4220 collected in TSBca were treated for 10 min with 1 μM R8K or with the peptide solvent. After this treatment, the indicated concentrations of eGFP-Ami11-CBD11 were added to the cells and lysis was evaluated spectrophotometrically. AMP solvent and endolysin buffer were added to the Control cells. Data of each curve are representative of 2 to 3 assays.

Gouveia *et al*.
Synthetic antimicrobial peptides as enhancers of the bacteriolytic action of staphylococcal phage endolysins

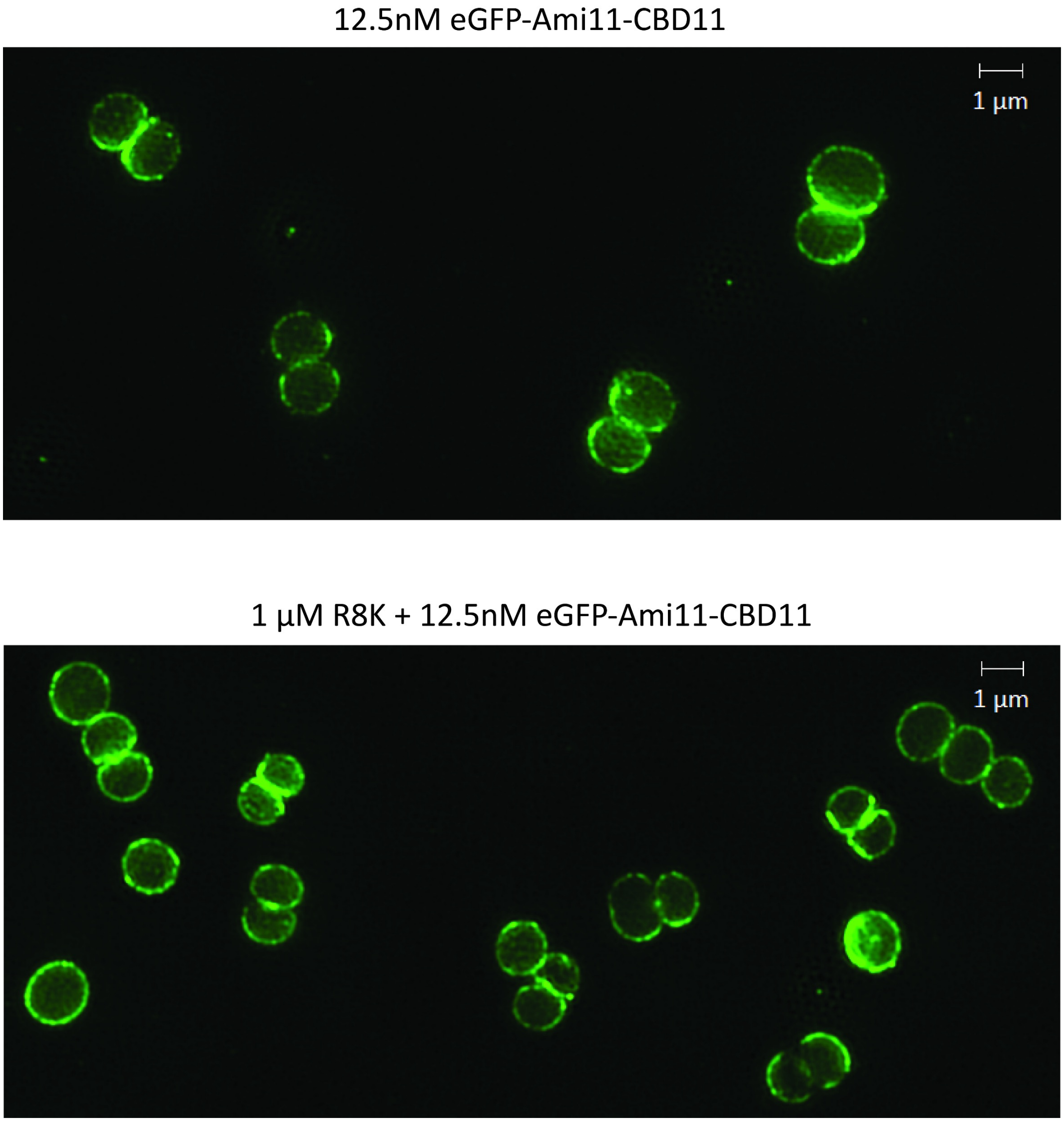

Figure S7. Pre-treatment with the AMP R8K does not alter eGFP-Ami11-CBD11 distribution pattern on *S. aureus* cell surface. Super-resolution Structured Illumination Microscopy (SIM) images of eGFP-Ami11-CBD11 binding to *S. aureus* RN4220 cells treated (bottom panel) or not treated (top panel) for 10 min with 1 µM R8K. Scale bar corresponds to 1 µm.

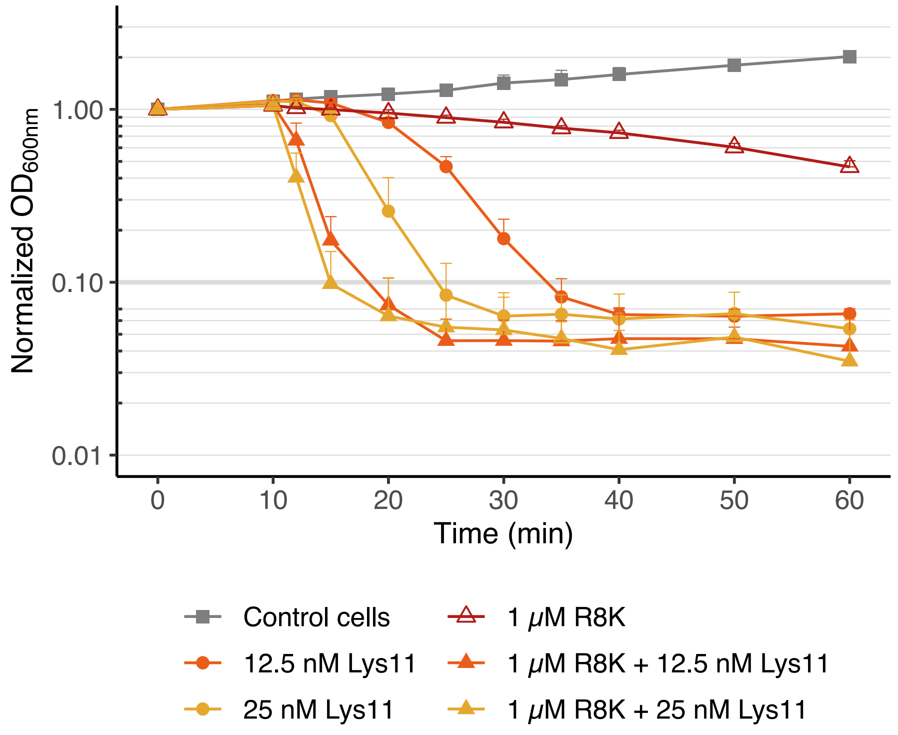

Figure S8. R8K stimulates Lys11-mediated lysis of tunicamycin-treated *S. aureus* cells. Log phase cells of strain RN4220 grown in presence of 50 ng/ml tunicamycin were collected in TSBca and then treated for 10 min with 1 μM R8K or with the peptide solvent. After this treatment, the indicated concentrations of Lys11 were added to the cells and lysis was evaluated spectrophotometrically. AMP solvent and endolysin buffer were added to the Control cells. The data of each curve represent means ± standard deviation from at least 3 independent experiments. For clarity, only mean + standard deviation is represented.
